# Trunk-Inspired SWCNT-Based Wrinkled Films for Highly-Stretchable Electromagnetic Interference Shielding and Wearable Thermotherapy

**DOI:** 10.1007/s40820-024-01454-w

**Published:** 2024-07-11

**Authors:** Xiaofeng Gong, Tianjiao Hu, You Zhang, Yanan Zeng, Ye Zhang, Zhenhua Jiang, Yinlong Tan, Yanhong Zou, Jing Wang, Jiayu Dai, Zengyong Chu

**Affiliations:** 1 College of Science, National University of Defense Technology, Changsha, 410073 People’s Republic of China; 2 Beijing Interdisciplinary Research Center, National University of Defense Technology, Changsha, 410073 People’s Republic of China; 3 School of Physics and Electronics, Hunan University, Changsha, 410082 People’s Republic of China

**Keywords:** Electromagnetic interference shielding, Single-walled carbon nanotubes, Wrinkles, Stretchable, Thermotherapy

## Abstract

Nowadays, the increasing electromagnetic waves generated by wearable devices are becoming an emerging issue for human health, so stretchable electromagnetic interference (EMI) shielding materials are highly demanded. Elephant trunks are capable of grabbing fragile vegetation and tearing trees thanks not only to their muscles but also to their folded skins. Inspired by the wrinkled skin of the elephant trunks, herein, we propose a winkled conductive film based on single-walled carbon nanotubes (SWCNTs) for multifunctional EMI applications. The conductive film has a sandwich structure, which was prepared by coating SWCNTs on both sides of the stretched elastic latex cylindrical substrate. The shrinking-induced winkled conductive network could withstand up to 200% tensile strain. Typically, when the stretching direction is parallel to the polarization direction of the electric field, the total EMI shielding effectiveness could surprisingly increase from 38.4 to 52.7 dB at 200% tensile strain. It is mainly contributed by the increased connection of the SWCNTs. In addition, the film also has good Joule heating performance at several voltages, capable of releasing pains in injured joints. This unique property makes it possible for strain-adjustable multifunctional EMI shielding and wearable thermotherapy applications.

**Supplementary Information:**

The online version contains supplementary material available at 10.1007/s40820-024-01454-w.

## Highlights

- Elephant trunk-inspired anisotropic wrinkling structures were formed on a sandwich-like conductive film through a controlled shrinking method.
- The wrinkled conductive network could withstand up to 200% tensile strain and exhibits a strain-enhanced electromagnetic interference shielding effectiveness when stretching parallel to the electric field polarization direction.

**Supplementary Information:**

The online version contains supplementary material available at 10.1007/s40820-024-01454-w.

## Graphical abstract

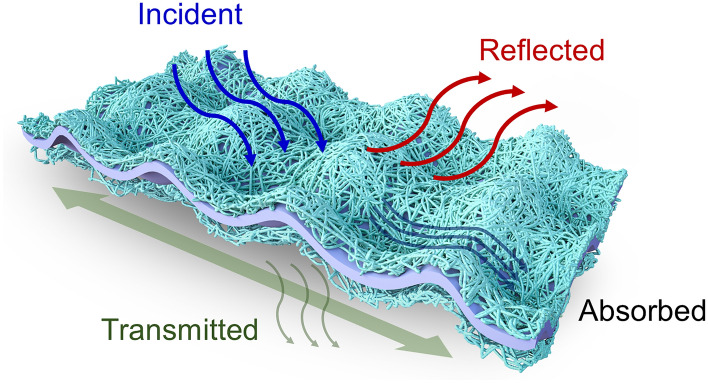

## Introduction

The vigorous development of next-generation flexible electronic devices, such as flexible displays and wearable human health monitoring systems, has brought great convenience to our daily lives [1, 2]. But at the same time, the widespread use of these electronic devices will produce massive electromagnetic interference (EMI), which then might lead to electronic equipment failure and adversely affect human health [3, 4]. Therefore, EMI is an essential problem in modern society, and the development of EMI shielding materials and EM wave absorption materials have become a research hotspot [5–12]. Traditional heavy metal-based EMI shielding materials have high-density, poor flexibility, and poor corrosion resistance and so cannot meet the requirements of flexible electronic devices [13]. From a wearable technology perspective, the right candidate material must have excellent deformation capabilities and excellent and stable EMI shielding efficiency under complex external loads such as stretching, bending, and twisting [14–17].

Stretchable conductors with excellent deformability and electrical conductivity are urgently needed for applications such as electronic skin, soft robotics, and EMI shielding materials [18–20]. To meet the requirements of these applications, stretchable conductors need to maintain stable electrical conductivity and structural robustness under complex deformation conditions. Large mechanical deformation of traditional conductors will have irreversible effects on electrical and structural stability. Much research has been carried out to develop stretchable conductors with strain-insensitive conductivity to solve this problem [21]. It is an effective method to make conductive materials into wavy shapes [22], serpentine mesh [23, 24], grid structures [25, 26], braided structures [27], etc., to promote the extension of intrinsically rigid materials during stretching. Li et al. [28] fixed the reduced graphene oxide film on the wavy substrate to obtain a stretchable conductive film, which could withstand 32.6% tensile strain and had a constant EMI shielding performance of 56.3 dB after multiple stretching cycles. Liu et al. [25] made a stable conductive cellular network structure from a spiral stretcher micro-coil and obtained a deformable conductive network after rubber encapsulation. Another effective method is to use an elastic substrate to prepare a composite material with a conductive layer and form a wrinkled structure through pre-stretching, thermal shrinkage, or solvent induction [29, 30]. Among them, creating wrinkle structures on the elastic substrate by the mechanical prestretch-release process is a simple and efficient method. The conductive material is deposited on a pre-stretched elastic matrix, which will buckle into a wrinkled shape on different length scales when the pre-stretching is released. Mechanical pre-stretching includes uniaxial and multi-axial stretching [31–34]. Dong et al. [29] prepared stretchable MXene-coated polyurethane fabric by an uniaxial pre-stretching and spraying method, and the unique wrinkle structure has a stable EMI shielding performance of about 30 dB under 50% tensile strain. Although the wrinkle structure has improved the tensile stability, the tensile strain is up to date limited to no more than 100%. Strong stretching with much higher tensile strain might lead to the slip or crack of the conductive network and affect the EMI shielding properties. Therefore, developing a stretchable conductor with high stretchability and strong structural stability is still a challenge.

A suitable elastic substrate must be selected to construct a stretchable conductor successfully. Latex is a polymer material with good tensile properties, which can be made into any shape and is a good choice for elastic substrates. To obtain conductive layers with high mechanical stability and stretchability, various active conductive materials such as reduced graphene oxide (RGO) [35, 36], Mxene [37, 38], silver nanowires (AgNWs) [39, 40], carbon nanotubes (CNT) [5, 41] and their composites have been widely used as conductive layers of stretchable conductors. Chen et al. [30] prepared a stretchable conductor with good tensile property and strain-invariant conductivity by constructing a folded MXene pattern on a flexible PDMS substrate. CNT can be regarded as a one-dimensional cylindrical nanomaterial composed of two-dimensional graphene sheets, which can be divided into single-walled carbon nanotubes (SWCNT) and multi-walled carbon nanotubes (MWCNT) according to the number of layers. Due to its unique physical properties, it has a wide range of applications in EMI shielding, microwave absorption, sensing, and other fields [5, 42–44].

Elephants drink water with their trunks, because they can expand their nasal volume to 164% by contracting the muscles in their nasal cavity. Elephant trunks can also be as flexible as the hands of primates, achieving versatility in grabbing fragile vegetation and tearing tree trunks. This is not only due to their muscles, but also because of their anisotropic folded skins. Inspired by the cylindrical trunks with highly folded skin, herein, we constructed a double-layered SWCNT conductive film with anisotropic wrinkle structures on the substrate of the elastic latex cylindrical balloons (DSWCNT@latex) through a simple three-dimensional shrinkage method. The anisotropic film has different EMI shielding properties at different testing directions. During the axial stretching process, when the stretching direction is parallel to the polarization direction of the electric field, the total shielding effectiveness (SE_T_) of the sample prepared with 0.12 wt% dispersion increases from 38.4 to 52.7 dB at 200% tensile strain. At the same time, based on the good electrical conductivity of SWCNT, the film also has good joule heating performance. The cylindrical base can be well conformal with the human wrist and arm joints, showing great potential in the field of smart wearable electronics for personal thermotherapy. Thermotherapy through the use of electric heating elements can accelerate blood circulation and relaxation of pain.

## Experimental Section

### Materials

Cylindrical latex balloons, 270 μm thick, were purchased from Hebei Youchuang Rubber Products Co., LTD, China. SWCNT dispersion (0.4 wt%) with 0.6 wt% sodium carboxymethylcellulose (CMC) was purchased from Shenyang Huijing Nanotechnology Co., LTD. Graphene oxide (GO) dispersion (1.0 wt%) was acquired from Changzhou Sixth Element Material Technology Co., LTD. Anhydrous ethanol (≥ 99.7 vol%) was purchased from Shanghai Titan Technology Co., LTD. Methyl silicone oil (RC0201) was purchased from LongXu Chemical Co., LTD.

### Preparation of DSWCNT@Latex Film

**Fig. 1:**
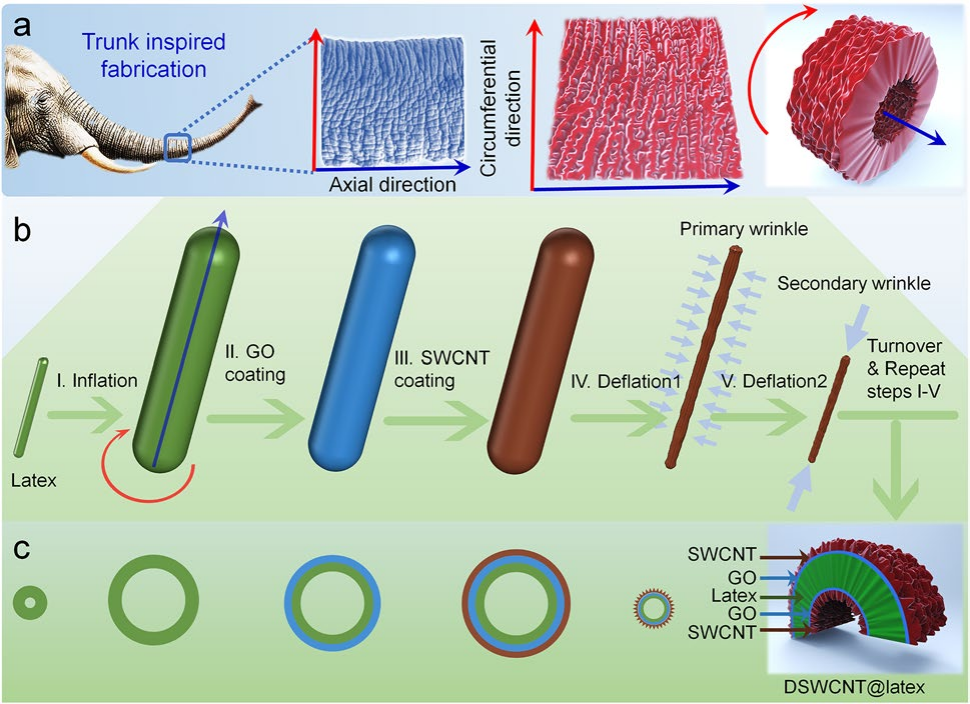
Schematic illustration of the trunk-inspired fabrication process. **a** Axial and circumferential directions of the cylindrical elephant trunk and its anisotropic surface wrinkles. **b** Schematic illustration of the fabrication process of the anisotropic microstructures of the primary and secondary wrinkles. **c** Schematic diagram of the cross-section morphology and the final sandwich-like double-layered cylindrical film

The cylindrical balloon was cleaned with anhydrous ethanol to remove oil and impurities on the surfaces. 0.4 wt% SWCNT dispersion was diluted to 0.08 wt%, and 0.12 wt% using deionized water. 1.0 wt% of GO dispersion was diluted to 1.0 mg mL^−1^ using anhydrous ethanol. The dispersions were ultrasonically mixed for 30 min. The preparation process is shown in Fig. 1. The cleaned substrate is inflated with a pre-strain (step I), and then a layer of GO alcohol dispersion is uniformly coated on the surface (step II). After drying, a layer of GO film is formed, and then a layer of SWCNT water dispersion is uniformly coated on the top of the GO film (step III). In the process, the GO layer acts as a good adhesion between the latex substrate and the SWCNT layer. Finally, anisotropic wrinkle structures could be induced by controlling the release of the circumferential strain and then the axial strain after deflations in step IV and step V sequentially. Then the inside of the balloon is turned over to the outside and inflated again with a pre-strain, and the above steps are repeated to obtain a double-layered SWCNT conductive film with anisotropic wrinkle structures on both sides of the elastic latex cylindrical balloons (DSWCNT@latex). The preparation process and detailed preparation parameters are shown in Video S1 and Table S1.

### Characterizations

Surface morphology and microstructure of the materials were analyzed using a transmission electron microscope (TEM, FEI Tecnai F20, USA) and scanning electron microscope (SEM, Hitachi S-4200, Japan and ZEISS Sigma 300, Germany). The mechanical properties were tested using a universal electronic testing machine (STD5000, Kunshan Lugong Precision Instrument, China). The resistivity was measured using a four-probe test system (M-6, Guangzhou Four Probe Technology, China). The resistance values during the stretching process were measured using a Tektronix multimeter (2750, Tektronix, USA). The chemical structures and functional groups of GO and SWCNT were characterized using a Fourier transform infrared spectrometer (Nicolet iS20, Thermo Scientific, American). Raman spectra were performed using the Horiba Raman confocal imaging microscope (LabRAM HR Evolution, Horiba, Japan) with a 532 nm laser.

A vector network analyzer (AV3672B, 41 Institute of China Electronics Technology Group, China) was used to test the EMI performance of the films under different tensile strains in the frequency range of 8.2–12.4 GHz (X-band). Two scattering parameters (*S*_11_ and *S*_21_) were obtained using the waveguide method. TE_10_ transverse wave was used as the signal source in the waveguide, in which the polarization direction of the electric field is parallel to the short edge of the waveguide. The reflection coefficient (*R*), transmission coefficient (*T*), absorption coefficient (*A*), total shielding effectiveness (SE_T_), reflection shielding effectiveness (SE_R_), and absorption shielding effectiveness (SE_A_) were calculated according to Eqs. (1–7) [45]. S_11_ represents the electromagnetic wave received by port 1 after the electromagnetic wave emitted from port 1 is reflected by the shielding material, and S_21_ represents the electromagnetic wave received by port 2 after the electromagnetic wave emitted from port 1 passes through the shielding material.

1 $$ R = \left| {S_{11} } \right|^{2} $$

2 $$ T = \left| {S_{21} } \right|^{2} $$

3 $$ A = 1 - R - T $$

4 $$ {\text{SE}}_{{\text{T}}} = - 10\log \left( T \right) $$

5 $$ {\text{SE}}_{{\text{R}}} = - 10\log \left( T \right) $$

6 $$ {\text{SE}}_{{\text{A}}} = - 10\log \left( T \right) $$

7 $$ {\text{SE}}_{{\text{T}}} {\text{ = SE}}_{{\text{R}}} {\text{ + SE}}_{{\text{A}}} {\text{ + SE}}_{{\text{M}}} $$

Multiple reflection shielding effectiveness (SE_M_) is usually ignored when SE_T_ > 15 dB [46]. For commercial uses, it is generally required that the SE_T_ of the shielding material is > 20 dB; that is, it can attenuate more than 99% of the electromagnetic wave [47].

The DC power supply (HCP-1022, Shenzhen Henghuiyuan Electronics, China) was charged at a specific voltage to measure the Joule heating performance. A multi-channel temperature controller (YP5008G, Yongpeng Instrument, China) was used to record the temperature change, and an infrared imager (Ti400, Fluke, USA) was used to observe the surface temperature distribution of the film.

## Results and Discussion

### Fabrication Parameters and Dimensional Characteristics

A curved elephant trunk is shown in Fig. 1a. There are obvious anisotropic wrinkles on the skin, large in axial and small in circumferential. Figure 1b and c shows the trunk-inspired schematic fabrication process of the DSWCNT@latex film. The cylindrical balloon substrate is inflated and coated with a layer of GO, then coated with a layer of SWCNT. The two-dimensional lamellar structure of GO is conducive to its adhesion to the rough substrate with physical interlocking and Van der Waals (VDW) forces, and the abundant oxygen-containing groups of GO are beneficial to the hydrogen bonding and VDW interaction with SWCNT film. Incorporating a layer of GO film promotes the adhesion between the SWCNT layer and the latex substrate. Subsequently, a wrinkled structure was prepared by slowly releasing the circumferential tensile strain first, while keeping the axial length unchanged (step IV) and then the axial tensile strain (step V). The primary small wrinkle was formed in the circumferential direction at step IV, and the secondary large wrinkle was formed in the axial direction at step V. SWCNT wrinkled films were coated on another side of the substrate by turning over the inside surface of the balloon to the outside and repeating the above steps. The final obtained sandwich-like double-layered film (Fig. 1c), named DSWCNT@latex, is black and matte (Fig. S1a).

The chemical structural characteristics of the materials were investigated with attenuated total reflection Fourier transform infrared spectroscopy (ATR-FTIR), Raman spectra and energy-dispersive X-ray spectroscopy (EDS) mapping. GO and SWCNT dispersions were dropped on the latex surface separately, and dried for comparisons. As shown in Fig. S2a–c, the typical peaks of GO film at 3401, 1715, 1620, and 1401 cm^−1^ are belong to the stretching vibrations of –OH, C=O, C=C, and C–OH groups, respectively. The typical peaks of SWCNT film embedded with CMC at 3430, 2970, 1630, and 1410 cm^−1^ are corresponding to the stretching vibrations of –OH, C–H, C=O, and C–OH groups, respectively. The typical peaks of DSWCNT0.12 film at 3390, 2920, 1640, and 1410 cm^−1^ are related to the stretching vibrations of –OH, C–H, C=O, and C–OH groups, respectively. All of them have abundant oxygen-containing groups. Raman spectra of GO and SWCNT are shown in Fig. S2d and e. In the Raman spectrum of GO, there are three obvious peaks located near 1350, 1580, and 2920 cm^−1^, corresponding to the bands of D, G, and 2D, respectively. G band represents the in-plane stretching vibration of carbon *sp*^2^ (graphitized carbon), and D band represents the structure of defects or amorphous in the carbon lattice. 2D band is caused by the out-of-plane vibrations of the lattice with few layers of graphite, and is considered to be characteristic peaks of the graphene structure. SWCNT film has a weak D band in addition to the sharp G and 2D bands, indicating that its structure has few defects. Because GO is very limited in the sample, the Raman spectrum of DSWCNT0.12 shown in Fig. S2f is similar to that of the raw material of SWCNT. EDS mapping of C, O elements of GO film and C, O, and Na elements of SWCNT film indicate the uniform distribution of the main elements of the raw materials (Fig. S1e–l).

**Fig. 2:**
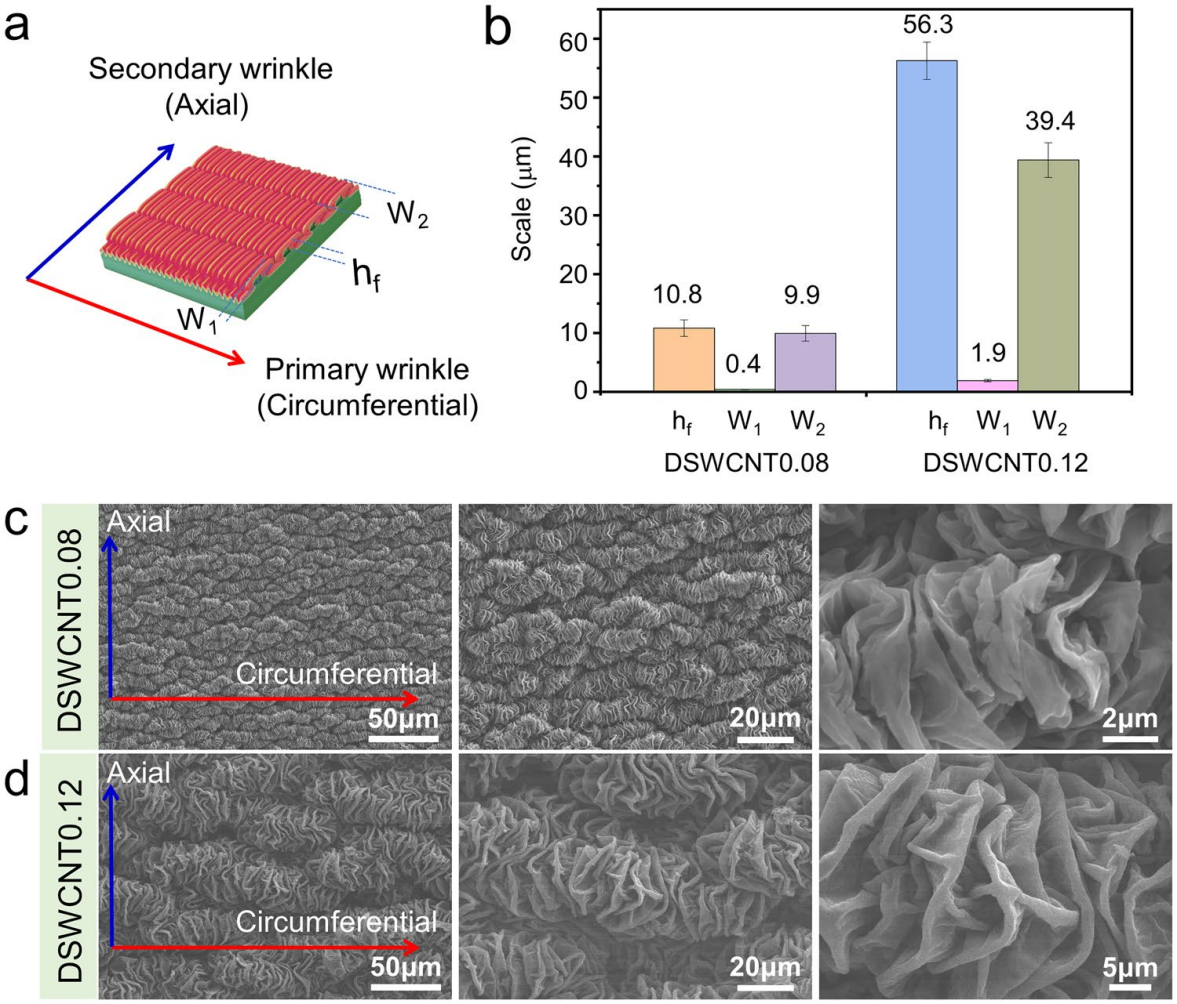
Microstructures of the wrinkled composite films. **a** Schematic illustration of the anisotropic microstructures of the primary and secondary wrinkles. **b** Dimensional characteristics of DSWCNT0.08 and DSWCNT0.12. SEM images of **c** DSWCNT0.08, and **d** DSWCNT0.12

Microstructures of the wrinkled films prepared with different SWCNT dispersions are shown in Fig. 2. As shown in Fig. 2a and b, the wrinkles in circumferential are defined as primary wrinkles with width denoted as *W*_1_; the wrinkles in axial are defined as secondary wrinkles with width denoted as *W*_2_; the film thickness is defined as *h*_f_. The sample was prepared from 0.08 wt% SWCNT is recorded as DSWCNT0.08, and the sample prepared from 0.12 wt% SWCNT is recorded as DSWCNT0.12. SWCNT used in this experiment is confirmed as having single-walled nanostructures (Fig. S1b), which is more conductive than MWCNT and so preferred for EMI shielding applications [34]. The microstructure of the winkled film demonstrates hierarchical and anisotropic surface morphology (Fig. 2c, d), well emulating the surface of the trunk skin. The primary wrinkles were first generated in the circumferential direction and the secondary wrinkles were subsequently generated in the axial direction. The dimensional characteristics of the primary and secondary wrinkles are different due to the different axial and circumferential strains (Fig. 2b).

The axial and circumferential shrinkage strains (*ε*_*l*_, *ε*_*d*_, respectively) applied to the film can be calculated according to Eqs. (8) and (9):

8 $$ \varepsilon_{l} = \Delta l/l_{0} $$

9 $$ \varepsilon_{d} = \Delta d/d_{0} $$

in which, Δ*l* = *l*_*f*_ − *l*_0_, is the length change in the axial direction; Δ*d* = *d*_*f*_ − *d*_0_, is the diameter change in the circumferential direction.

In this experiment, the axial shrinkage strain during the preparation process is about 200%, and the circumferential shrinkage strain is about 500%. Based on the statistics of the wrinkle width and film thickness via SEM observations (Fig. S3), the huge different shrinkage strains in different directions, as well as the different shrinking sequence priority, make a great deviation of *W*_1_ and *W*_2_ (0.4 and 9.9 μm for DSWCNT0.08; 1.9 and 39.4 μm for DSWCNT0.12, respectively, as shown in Fig. 2b). The wrinkle widths of DSWCNT0.12 is much larger than those of DSWCNT0.08, which are mainly due to the much higher film thickness of DSWCNT0.12 (56.3 μm) than that of DSWCNT0.08 (10.8 μm). Generally speaking, higher thickness leads to a larger wrinkle wavelength or width [48], so the wrinkle width can be easily adjusted via the film thickness or the concentration of SWCNT dispersion.

### Mechanical and Electrical Properties

**Fig. 3:**
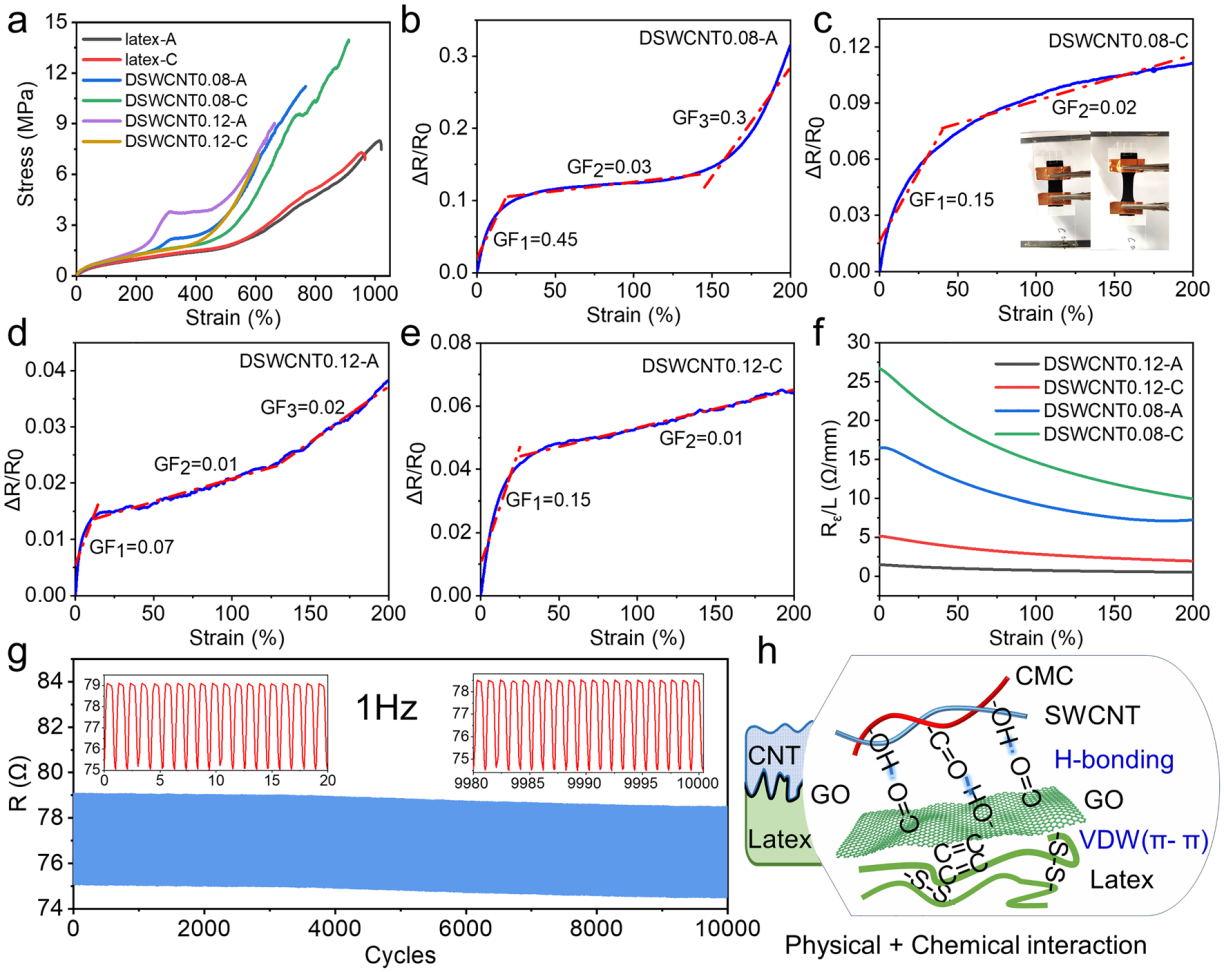
Mechanical and electrical properties of the wrinkled composite films. **a** Tensile strain curves of the latex substrate, DSWCNT0.08, and DSWCNT0.12. **b** Δ*R*/*R*_0_ − *ε* curve of DWSCNT0.08 during axial stretching. **c** Δ*R*/*R*_0_ − *ε* curve of DWSCNT0.08 during circumferential stretching (inset: optical photos of DWSCNT0.12 during tensile test). **d** Δ*R*/*R*_0_ − *ε* curve of DWSCNT0.12 during axial stretching. **e** Δ*R*/*R*_0_ − *ε* curve of DWSCNT0.12 during circumferential stretching. **f** Resistance per unit length of DWSCNT@latex during axial and circumferential stretching. **g** Resistance cycling stability of DWSCNT0.12 during 10,000 cycles of axial stretching. **h** Diagram of the interlayer interaction

Mechanical properties of the films were further investigated using uniaxial stretching under ambient conditions. Tensile strain curves were measured in different stretching directions (axial and circumferential) to compare the elongation at the break state, as shown in Fig. 3a. It was observed that compared with the simple latex substrate, the elongation at the break of the wrinkled composite films decreases with the increase of the concentration because there are residual compressive strains in the elastic substrate of the composite films. The higher the concentration, the thicker the film layer, and the larger the residual strain. But the elongation at break of SWCNT0.12 can be still as large as 600%. It has better stretching capabilities than most stretchable composites [14, 20, 26, 49–53] (Table S2). This is attributed to the good elastic strain of the latex substrate and the high deformability of the wrinkle structure. The polymer chains of the latex are entangled with each other or crosslinked with –S–S– bonds. Upon stretching, the curly molecular chains can be straightened to a large state; upon relaxing, it can shrink back to the original shape. So the latex has good elasticity.

The tape stripping test proved that the interfacial bonding between the wrinkled SWCNT layer and the latex substrate is very strong (Fig. S4). The sample was glue-fixed on the glass sheet on one side and then glue-fixed with a strip of 30 mm × 30 mm tape on the surface (Fig. S4a). The tape was peeled off from the surface at a force of about 3–4 N (Fig. S4b). The surface of the tape was very clear without visible peeling species even after five times of stripping (Fig. S4c), and the surface of the wrinkled sample remains its original morphology (Fig. S4d). The rough mortise and tenon structure of the latex substrate (Fig. S1b) enhanced the physical interfacial bonding. The cross-section SEM image indicates that the film layer and the latex substrate are firmly bonded, showing an interlocking structure (Fig. S4e, f). During the preparation of the film layer, SWCNT will penetrate into the rough groove structure and be tightly squeezed during the contraction of the substrate.

The surface resistivity of the film was measured using a four-probe square resistance meter (Fig. S5a). The average surface resistivity of DSWCNT0.08 is 11.49 Ω cm, and that of DSWCNT0.12 is 3.36 Ω cm. The higher the concentration of SWCNT dispersion, the better the electrical conductivity. The sample was cut into a rectangle of 30 mm × 10 mm, with two ends fixed on a glass sheet along the long side. The copper foil was connected to the two ends to measure the resistance change during the stretching process (Fig. S5b). The variation of the relative resistance change (Δ*R*/*R*_0_) with the tensile strain (*ε*) was calculated and the results are shown in Fig. 3b–e.

Since, the axial compression strain is no more than 200%, the highest tensile strain up to 200% was studied here. It was observed that Δ*R*/*R*_0_ gradually increases with the progress of stretching, whether it is axial or circumferential stretching. The gauge factor (GF, the ratio of Δ*R*/*R*_0_ to *ε*) can be used to measure the strain-sensitivity of conductive materials [33]. Whether in axial or circumferential stretching, GF is relatively higher (0.07–0.45) at the beginning stage (*ε* is lower than about 25%). From the perspective of wrinkle structure, it is because of the sudden separation of the structures in contact with each other. GF is as low as 0.01–0.03 at the middle stage (*ε* is in the range of 25%–150%), indicating an excellent strain-insensitive property. The resistance will grow faster at the end of the axial stretching because the wrinkle structure is straightened (Fig. 3b).

**Fig. 4:**
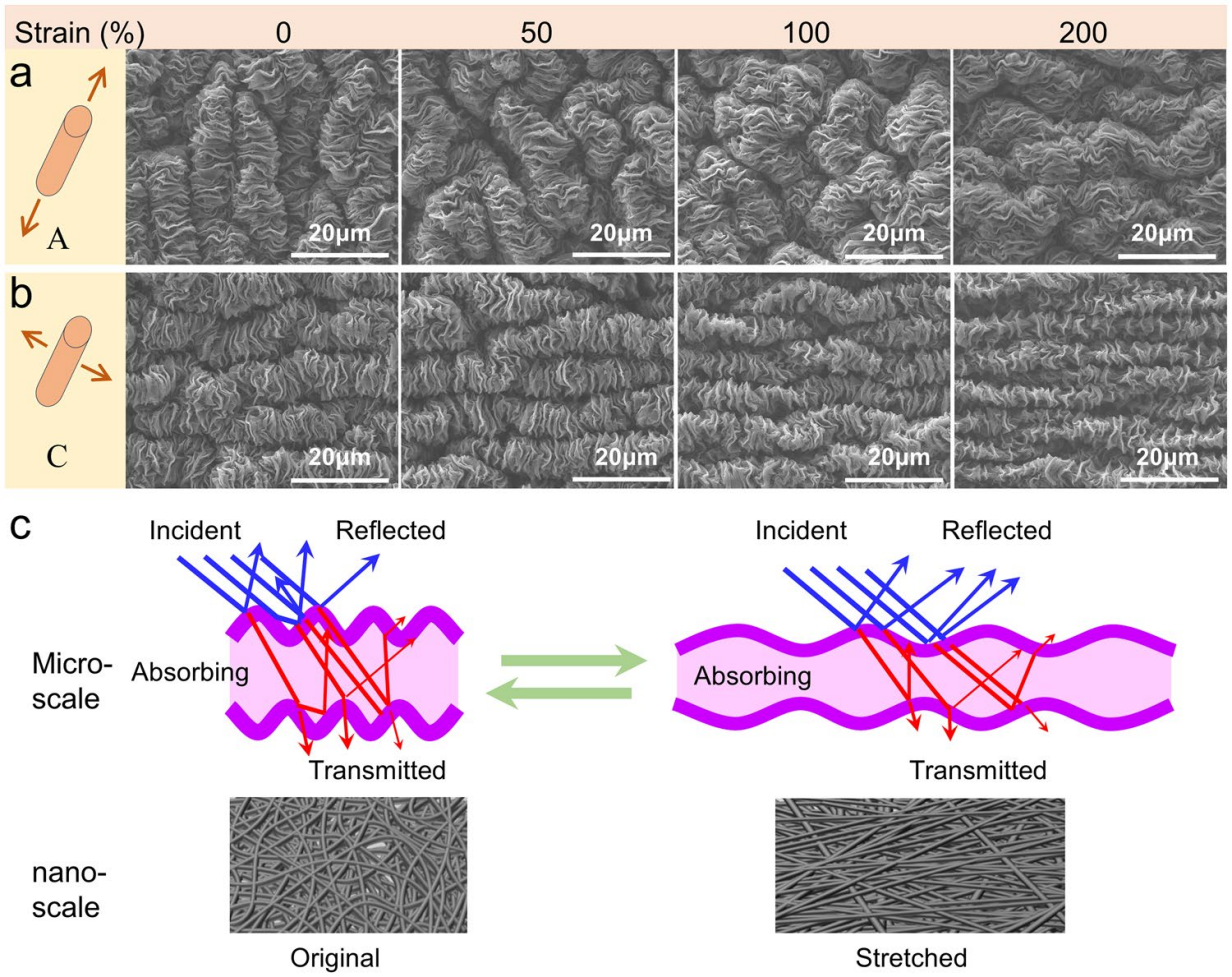
Microstructure evolution with the progress of stretching. SEM images of DSWCNT0.08 with the process of stretching in **a** axial, and **b** circumferential directions. **c** Schematic illustration of the structure evolution at the micro- and nanoscale

The microstructure evolution with the progress of stretching was studied using SEM analysis. The results are shown in Fig. 4. In the process of axial stretching (Fig. 4a), the secondary wrinkles were straightened and became smooth gradually, whereas the primary wrinkles changed the direction to the axial at the tensile strain of 200%; in the process of circumferential stretching (Fig. 4b), the primary wrinkles expand progressively with an obvious increase of the wrinkle width, whereas the secondary wrinkles maintain their directions with a gradual decrease of the wrinkle width.

In addition to the index of GF, the resistance per unit length along the tensile direction (*R*_*ε*_/*L*) can present some more insights into the conductive film. The results are shown in Fig. 3f. The axial resistance per unit length is lower than that of the circumferential resistance because the circumferential pre-strain is much higher and its original conductive path is longer. In addition, with the progress of stretching, the overall resistance will increase, but the resistance per unit length demonstrates a decreasing trend, whether it is axial or circumferential. This is because the interwoven SWCNT conductive networks will become more conductive during the stretching process (Fig. 4c), which is very common for the weave fabrics. To evaluate the stability of the film during repeated stretching, the resistance change between the tensile strains of 0–50% was tested up to 10,000 stretching cycles, as shown in Fig. 3g. The resistance is repeatable. After 10,000 stretching cycles, the variation of the resistance is about 0.5 Ω, indicating that the interwoven network structure of SWCNT has excellent recovery performance and the film remains stable as a whole with good fatigue resistance. CMC is intertwined with SWCNT into a tight network. In addition to the interwoven network of SWCNT (Fig. S4f), the strong interlayer bondings between the films of SWCNT, GO, and latex (Fig. 3h) play an important role. It includes the physical interlocking between GO and the rough surface of latex substrate, as well as the chemical interaction of hydrogen bonding between GO and CMC, π–π VDW forces between GO and SWCNT, and VDW forces between GO and latex.

### Electromagnetic Interference Shielding Performance

**Fig. 5:**
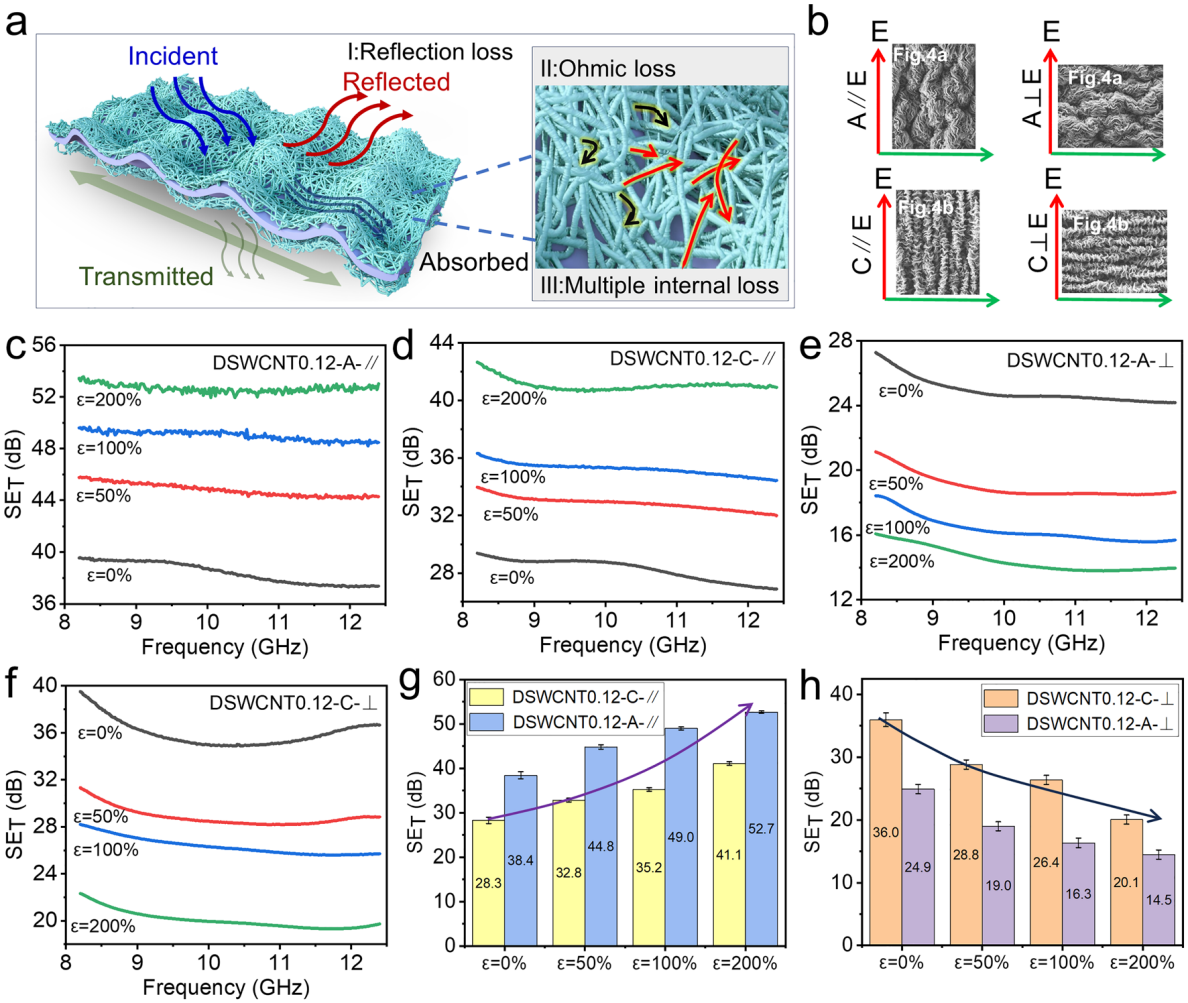
EMI shielding performance of the wrinkled composite films. **a** Schematic diagram of the EMI shielding mechanism. **b** Schematic diagram of the axial (A) or circumferential (C) stretching direction of the film parallel (//) or perpendicular (⊥) to the polarization direction of the electric field. **c** SE_T_ of stretched DSWCNT0.12 when A//E. **d** SE_T_ of stretched DSWCNT0.12 when C//E. **e** SE_T_ of stretched DSWCNT0.12 when A⊥E. **f** SE_T_ of stretched DSWCNT0.12 when C⊥E. **g** Average SE_T_ at of stretched DSWCNT0.12 when the stretching direction is parallel to the polarization direction of the electric field. **h** Average SE_T_ of stretched DSWCNT0.12 when the stretching direction is perpendicular to the polarization direction of the electric field

Excellent stretchability and good electrical conductivity lay a good foundation for flexible EMI shielding applications. Figure 5 shows the shielding performance of the wrinkled composite film. As a conductive shielding material, when the incident electromagnetic waves go to the surface of DSWCNT@latex (Figs. 5a and 4c), most of them will be reflected directly due to the impedance mismatch between the air and SWCNT film. Subsequently, part of the electromagnetic wave enters the network structure of SWCNT film and interacts with the high-density electron carrier, resulting in a large number of ohmic losses of the electromagnetic wave energy and the attenuation of heat energy. In addition, the multiple reflections inside the SWCNT network structure also contribute to the dissipation of electromagnetic waves, thus improving the overall shielding effect. Leaving only a tiny part of the electromagnetic wave passing through the film [54]. A vector network analyzer was used to measure the EMI shielding properties in the X-band (Fig. S6a), which can tell each amount of the reflected, absorbed, and transmitted energy. During the measurement, the film was stretched and fixed perpendicular to the propagation direction of the electromagnetic wave. However, two issues guide the measurement into four cases. Namely, (1) the film is anisotropic due to the anisotropic assembly of the wrinkles, so the stretching can be divided into axial stretching (A) and circumferential stretching (C), as shown in Fig. 4; (2) the electromagnetic wave transmitted through the waveguide is TE_10_ transverse wave (Fig. S6b), in which the polarization direction of the electric field is parallel to the short edge of the waveguide, so the stretching direction can be parallel (∥) or perpendicular (⊥) to the polarization direction of the electric field. Therefore, as shown in Figs. 5b and S6c, d, there are overall four different cases, A∥E, A⊥E, C∥E, and C⊥E.

For the same sample, such as DSWCNT0.12 shown in Fig. 5c–f, the EMI shielding performances of the four cases are different from each other. Figure 5c–f corresponds to the total EMI shielding effectiveness (SE_T_) of DSWCNT0.12 at the testing conditions of A∥E, C∥E, A⊥E, and C⊥E, respectively. When the stretching direction is parallel to the polarization direction of the electric field (Fig. 5c, d, g), SE_T_ increases with the progress of stretching. The initial SE_T_ of DSWCNT0.12 is about 38.4 dB and reaches 52.7 dB at 200% strain when stretched in axial (Fig. 5c). During the circumferential stretching (Fig. 5d), the initial SE_T_ is about 28.3 dB and reaches 41.1 dB at 200% strain. Surprisingly, when the stretching direction is parallel to the polarization direction of the electric field, all the EMI shielding performances increase gradually with the progress of stretching (Fig. 5g). It indicates a good strain-enhanced EMI shielding performance. This is contributed by the enhanced orientation of the SWCNT upon stretching (Fig. 4c), which can increase EMI shielding performance when its enhanced orientation is parallel to the polarization direction of the electric field. In addition, the whole EMI shielding performance in the axial direction is higher than that in the circumferential direction, which follows the low resistance per unit length in axial shown in Fig. 3f. That means that the secondary large winkles make the axial direction more conductive.

On the other hand, when the stretching direction is perpendicular to the polarization direction of the electric field (Fig. 5e, f, h), the changing behavior of EMI shielding performance is opposite. SE_T_ decreases with the progress of stretching. The initial SE_T_ of DSWCNT0.12 is about 24.9 dB, and decreases to 14.5 dB at 200% strain when stretched in axial (Fig. 5e); the initial SE_T_ is about 36 dB and decreases to 20.1 dB at 200% strain when stretched in circumferential (Fig. 5f).

**Fig. 6:**
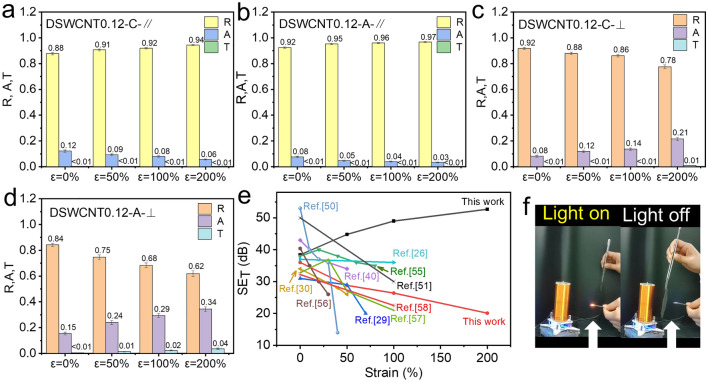
Contribution of R, A, and T to EMI shielding performance of the wrinkled composite films. **a** R, A, and T of DWSCNT0.12 when the axial stretching direction is parallel to the polarization direction of the electric field. **b** R, A, and T of DWSCNT0.12 when the circumferential stretching direction is parallel to the polarization direction of the electric field. **c** R, A, and T of DWSCNT0.12 when the axial stretching direction is perpendicular to the polarization direction of the electric field. **d** R, A, and T of DWSCNT0.12 when the circumferential stretching direction is perpendicular to the polarization direction of the electric field. **e** Comparison of EMI shielding performance of DSWCNT0.12 with recently reported other stretchable shielding materials. **f** Digital pictures of wireless power transmission system (Tesla coil): (left) without shield, and (right) with DSWCNT0.12

Opposite to the parallel states shown above, when the stretching direction is perpendicular to the polarization direction of the electric field, all the EMI shielding performances decrease gradually with the progress of stretching (Fig. 5h). However, this behavior is a very common characteristic of the shielding materials. Figure 6e lists the shielding properties of stretchable EMI shielding materials reported in recent years [26, 29, 30, 40, 50, 51, 55–58]. It can be observed that the shielding performances of other reported stretchable materials will decrease with the progress of stretching, similar to DSWCNT0.12 at A⊥E and C⊥E states, only DSWCNT0.12 at A∥E and C∥E states could increase with the progress of stretching, and could reach above 50 dB at 200% strain. To be pointed out, the state of A∥E is not associated with the state of C⊥E, because they are in different stretching states and means two different materials (Fig. 5b). For only one anisotropic sample of DSWCNT0.12, we can make it stain-enhanced to SE_T_ > 50 dB, or make it strain-weakened to SE_T_ < 15 dB, depending the stretching direction and the placing direction. One material could lead to four levels of EMI shielding performance, marking a great prospective application.

The variation of EMI shielding performance of DSWCNT0.08 upon stretching is similar to that of DSWCNT0.12 (Figs. S7, S8). However, the whole performance is much lower than that of DSWCNT0.12 (Figs. S7, S8), which is reasonable because its conductivity is much lower due to the lower loading of SWCNT (Figs. 2a, 3f). To analyze the shielding mechanism of the wrinkled film, R, A, and T were calculated and are shown in Fig. 6. It is anticipated that the shielding effectiveness of the wrinkled film is dominated by the reflection loss. When the stretching direction is parallel to the polarization direction of the electric field (Fig. 6a, b), R increases with the progress of stretching and SE_T_ increases simultaneously. The increase of R is mainly due to the increase of electrical conductivity per unit length along the stretching direction, which enhances the reflection ability of electromagnetic waves. On the other hand, when the stretching direction is perpendicular to the polarization direction of the electric field (Fig. 6c, d), R decreases with the progress of stretching and SE_T_ decreases simultaneously. This is because the electrical conductivity per unit length will increase along the stretching direction, while the electrical conductivity per unit length will decrease perpendicular to the stretching direction (i.e., parallel to the polarization direction of the electric field), due to the reorientation of the wrinkles. In a word, the electrical conductivity per unit length parallel to the polarization direction of the electric field determines the whole reflection ability, thus dominating the whole shielding effectiveness.

The EMI shielding effectiveness of DSWCNT0.12 is demonstrated visually using a Tesla coil and a light-emitting diode (LED), as shown in Fig. 6f and Video S2. It can be observed that the LED is lit up near the assembled Tesla coil after turning on the power supply. When the film is inserted between the coil and the bulb, the bulb goes out immediately.

The EMI shielding performance after 10,000 stretching cycles is shown in Fig. S10a, the shielding performance of DSWCNT0.12 is slightly increased when it is tested in the state of A∥E after 10,000 axial stretching. Because there is an unrecovered elastic strain in the sample after repeated stretching, the length of the sample has a slight increase.

### Joule Heating and Thermotherapy Performance

In addition to EMI shielding applications, there is a high demand for integrated heating devices to meet the rapid development of flexible wearable thermotherapy devices [59, 60]. The wrinkled film has good electrical conductivity and stretchability and can be used as a stretchable Joule heater. According to the Joule heating principle, the heat generated when an electric current passes through a conductor can be shown by Eq. (10) [39].

10 $$ Q = UIt = I^{2} Rt = U^{2} t/R $$,

where *Q, U, I, R*, and *t* represent the generated Joule heat, applied voltage, current, conductor resistance, and working time, respectively.

**Fig. 7:**
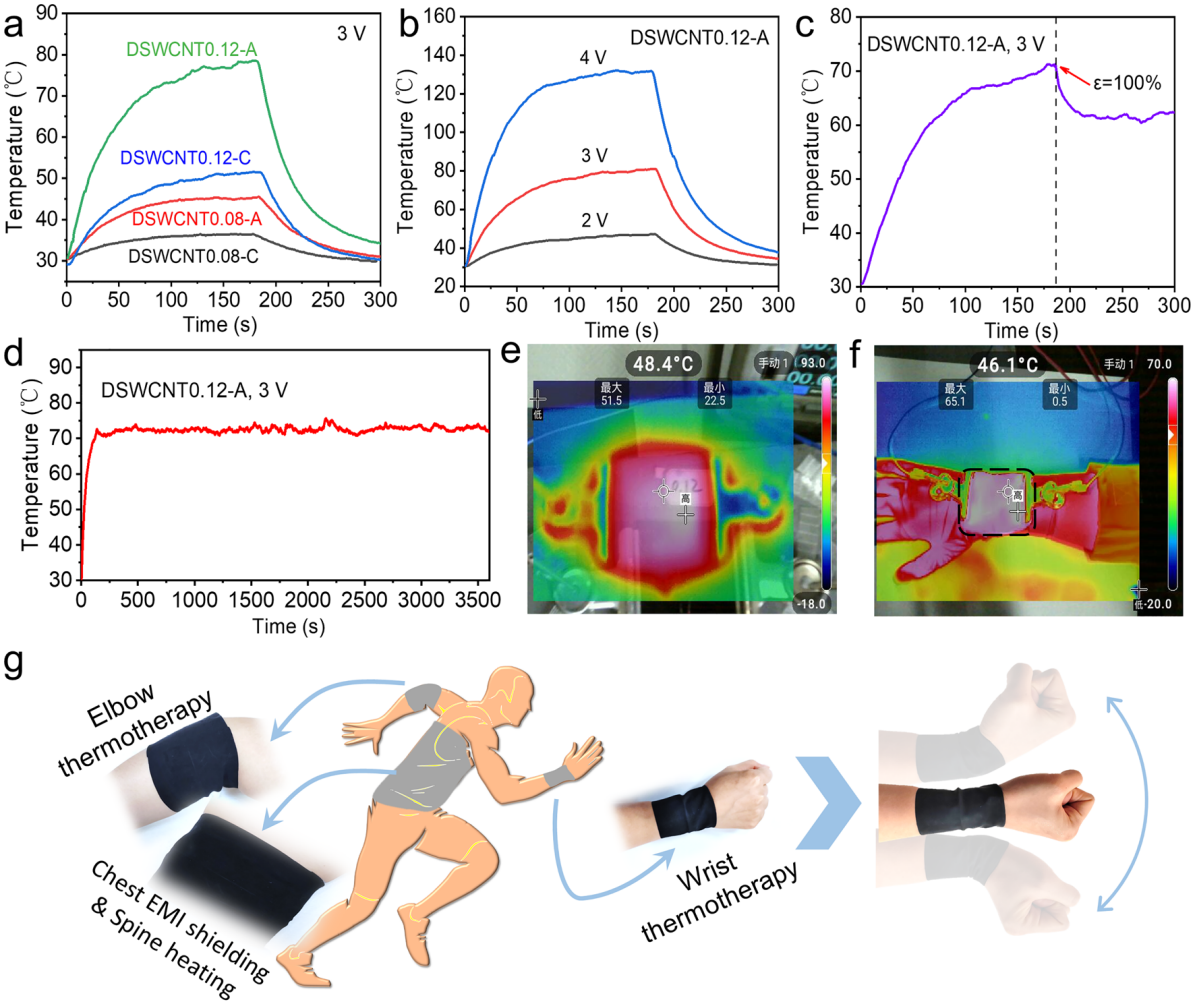
Joule heating and thermotherapy performance of the wrinkled composite films. **a** Temperature changes of DSWCNT0.08 and DSWCNT0.12 under 3 V in axial or circumferential. **b** Temperature changes of DWSCNT0.12 under 2 V, 3 V, and 4 V in axial. **c** Temperature changes of DWSCNT0.12 stretched in axial. **d** Temperature changes of DWSCNT0.12 under 3 V in axial for 1 h. **e** Infrared image of DWSCNT0.12 under 3 V. **f** Infrared image of DSWCNT0.12 in personal wearable thermotherapy. **g** Application demonstrations of the cylindrical film which is conformal to the elbow, wrist, and chest for EMI shielding or thermotherapy

Here, we cut the film into a 30 mm × 30 mm square, connect it to the DC power supply, and record the surface temperature change with a temperature tester, as shown in Fig. 7. The heating temperatures of different samples under the same voltage and in different directions were compared, as shown in Fig. 7a. It can be observed that, under a voltage of 3 V, the saturation temperature of DSWCNT0.08 is about 36 °C when the voltage is applied in circumferential, and reached 46 °C when the voltage was applied in axial. For DSWCNT0.12, the saturation temperature is about 51 °C, when the voltage is applied in circumferential and 78 °C when the voltage is applied in axial. The higher the electrical conductivity, the higher the saturation temperature under the same voltage. As expected, there is the highest temperature for DSWCNT0.12 in the axial because it has the best electrical conductivity.

The surface temperature change of DSWCNT0.12 with the variation of voltage in axial was further studied, as shown in Fig. 7b. The saturation temperature is 47, 81, and 130 °C under 2, 3, and 4 V, respectively. The higher the voltage, the higher the saturation temperature. At the same time, the influence of stretching on the surface temperature was also studied, as shown in Fig. 7c. Upon stretching to 100% (the length is doubled), the temperature (about 70 °C) will suddenly drop to a new equilibrium temperature (about 60 °C) due to the sharp increase of the whole resistance. Therefore, the heating temperature can be regulated in a certain range by simply stretching the film.

The film can maintain good temperature stability during a 1 h-long-term test, as shown in Fig. 7d. There is a slight fluctuation of the saturation temperature but is limited to ± 3 °C. The infrared thermal imager indicates a uniform temperature distribution (Fig. 7e, Video S3). The good Joule heating property of DSWCNT0.12 makes it suitable for wearable heating devices, which can serve as thermotherapy for joint injections, easily heating to above 40 °C to relieve pains (Fig. 7f). To be pointed out, the cylindrical macrostructure is easily conformal to the wrists, arms, and elbows (Fig. 7g, h), moving up and down as freely as the elephant trunk does. In addition, the film can also be used as a flexible deicing device at low voltages. For example, the ice on the film could melt and fall off within the 60 s under 5 V heating (Fig. S11). So, the trunk-inspired SWCNT-based wrinkled films have great potential for multifunctional wearable devices such as highly-stretchable electromagnetic interference shielding and wearable thermotherapy.

As wearable devices, air permeability should be considered so as to improve the comfort of wearing. Latex balloon itself has a very limited air permeability, so we used a fine needle with a diameter of 0.1 mm to pierce holes in the film at 5 mm intervals to improve the permeability. As shown in Video S4, air bubbling with good air permeability is successfully demonstrated after punching. At the same time, the mechanical properties, tensile electrical properties (Fig. S12), and EMI shielding properties (Fig. S10b) of the punched film are well kept.

The water-resistance of the film is also a key issue for real applications [61], because there are many hydrogen bonding between the layers. Our results showed that the sample could withstand ultrasonic treatment in water for 60 min (Fig. S13). When soaked in water for one day, dried, and then soaked and dried for three cycles, the microstructure remain stable with no significant change. But longer soaking or longer ultrasonic treatment may destroy the morphologies.

In order to improve the longer stability of the sample in a humid environment, we increased its hydrophobic property by vacuum impregnating methyl silicone oil onto the surface. The hydrophobic performance was well improved so that droplets can roll down freely from the surface, as shown in Video S5. As observed from the optical and SEM images of the sample impregnated with methyl silicone oil, shown in Fig. S14, there is no worsening effect on the microstructures. EDS mapping indicates the uniform distribution of Si element. The tensile electrical property and EMI shielding property are also well kept (Figs. S14d and S10c), indicating that it is a good method to improve the waterproof performance of the films.

## Conclusion

In summary, anisotropic wrinkled SWCNT films were constructed on the surface of an elastic latex substrate by a three-dimensional shrinkage process, and a highly stretchable composite film with a sandwich-like structure was prepared. The film has good tensile properties and its resistivity per unit length can be improved along the stretching direction, due to the reorganization of the wrinkles and the nanotubes. It can be used as a stretchable EMI shielding film with unique strain-enhancing or strain-weakening properties, depending on the stretching direction and its relation to the polarization direction of the electric field. One material could lead to four levels of EMI shielding performance, marking a great prospective application. In addition, the unique conductive network shows excellent Joule heating performance. The cylindrical curved structure makes it well conformal to the human wrist and other joints. So, all the above properties the film presents make it many perspectives for a multifunctional wearable device with electromagnetic protection and efficient thermal energy management.

## Supplementary Information

Below is the link to the electronic supplementary material.

Supplementary file1 (DOCX 7867 KB)

Supplementary file2 (MP4 13266 KB)

Supplementary file3 (MP4 4790 KB)

Supplementary file4 (MP4 4286 KB)

Supplementary file5 (MP4 1752 KB)

Supplementary file6 (MP4 1329 KB)
