# Supplementary material for: Trunk-Inspired SWCNT-Based Wrinkled Films for Highly-Stretchable Electromagnetic Interference Shielding and Wearable Thermotherapy
Source: Nanomicro Lett. 2024 Jul 11;16:243. doi: 10.1007/s40820-024-01454-w (PMC11239633; doi:10.1007/s40820-024-01454-w)
Supplement: Supplementary file 1 — Supplementary file1 (DOCX 7867 KB) [file 40820_2024_1454_MOESM1_ESM.docx]

Supporting Information for

**Trunk-Inspired SWCNT-Based Wrinkled Films for Highly-Stretchable Electromagnetic Interference Shielding and Wearable Thermotherapy**

Xiaofeng Gong ^1,†^, Tianjiao Hu^1,†^, You Zhang^1^, Yanan Zeng^3^, Ye Zhang^1^, Zhenhua Jiang^1^, Yinlong Tan^2,^*, Yanhong Zou^3^, Jing Wang^1^, Jiayu Dai^1,^* and Zengyong Chu^1,^*

^1^ College of Science, National University of Defense Technology, Changsha 410073, P. R. China

^2^ Beijing Interdisciplinary Research Center, National University of Defense Technology, Changsha 410073, P. R. China

^3^School of Physics and Electronics, Hunan University, Changsha 410082, P. R. China

^†^Xiaofeng Gong and Tianjiao Hu contributed equally to the work.

*Corresponding authors. E-mail: [tanyinlong15@nudt.edu.cn](mailto:tanyinlong15@nudt.edu.cn) (Yinlong Tan); [jydai@nudt.edu.cn](mailto:jydai@nudt.edu.cn) (Jiayu Dai); [chuzy@nudt.edu.cn](mailto:chuzy@nudt.edu.cn) (Zengyong Chu)

**Supplementary Tables and Figures**

# Table S1 Construction parameters of the films

| Fabrication Parameters | DSWCNT0.08 | DSWCNT0.12 |
| --- | --- | --- |
| d_1_ (cm) | 6.4 | 6.4 |
| d_2_ (cm) | 42.0 | 41.6 |
| d_3_ (cm) | 6.8 | 7.2 |
| d_4_ (cm) | 41.5 | 41.0 |
| d_5_ (cm) | 7.6 | 8.0 |
| l_1_ (cm) | 20.0 | 20.0 |
| l_2_ (cm) | 66.0 | 66.0 |
| l_3_ (cm) | 21.5 | 22.0 |
| l_4_ (cm) | 65.0 | 65.0 |
| l_5_ (cm) | 21.5 | 22.0 |

d_1_:circumference of the uninflated substrate,

d_2_: circumference of the substrate after the first inflation,

d_3_: circumference of the substrate after the first deflation,

d_4_: circumference of the substrate after the second inflation,

d_5_: circumference of the substrate after the second deflation,

l_1_:length of the uninflated substrate,

l_2_: length of the substrate after the first inflation,

l_3_: length of the substrate after the first deflation,

l_4_: length of the substrate after the second inflation,

l_5_: length of the substrate after the second deflation.

# Table S2 Comparison of the mechanical properties of related works

| Composites | Breaking elongation (%) | Tensile strength(MPa) | Thickness(mm) | Refs. |
| --- | --- | --- | --- | --- |
| PU-AgNW/CFF fabric | 15 | 9.6 | 0.36 | [S1] |
| TaS_2_ film | 5 | 125 | 0.003 | [S2] |
| MFEA/AgNWs film | 280 | 3.7 | 5 | [S3] |
| PEDOT:PSS/Li-TFSI/XSB film | 200 | 10 | 0.2 | [S4] |
| EMA/ TPO/RGO film | 653 | 7.1 | 1 | [S5] |
| LM/PDMS composite mesh | 150 | 0.4 | 0.4 | [S6] |
| AMLM/Ecoflex | 600 | 0.3 | 0.2-0.78 | [S7] |
| LM/PDMS lattice | 180 | 2.6 | 13 | [S8] |
| DSWCNT/Latex film | 660 | 9 | 0.27 | This work |

# Table S3 Comparison of EMI shielding performance of related works

| Composites | Preparation method | Thickness  (mm) | EMI(dB) | | Refs. |
| --- | --- | --- | --- | --- | --- |
|  |  |  | Original | Stretched (tensile strain) |  |
| MFEA/AgNWs film | polycondensation | 5 | 53 | 36(30%) | [S3] |
| PEDOT:PSS/XSB film | evaporation | 0.2 | 40.9 | 30.9(100%) | [S4] |
| PDMS/LM composite mesh | cast molding and mechanical sintering | 0.4 | 35 | 42(60%) | [S6] |
| LM/PDMS lattice | 3D printing | 3 | 37 | 36(100%) | [S8] |
| MXene/TPU film | uniaxial prestretching | >0.3 | 31 | 29(50%) | [S9] |
| Steel wool/rubber film | cast molding | 5 | 38 | 35(80%) | [S10] |
| MXene/PDMS film | thermal annealing and spin-coating | - | 30 | 26(50%) | [S11] |
| Cu mesh/PDMS film | self-forming and electroplating | - | 40.4 | 26(30%) | [S12] |
| MXene organohydrogel | solvent displacement | 1 | 32.8 | 21(100%) | [S13] |
| AgNWs/PDMS film | pre-stretching and spray coating | 0.2 | 32.2 | 22.5(100%) | [S14] |
| CNT/TPU film | microwave sintering | 2 | 35.5 | 26.5(100%) | [S15] |
| DSWCNT/Latex film | 3D pre-stretching and coating | 0.3 | 38.4 | 52.7(200%) | This  work |
|  |  |  | 36 | 20.1(200%) |  |


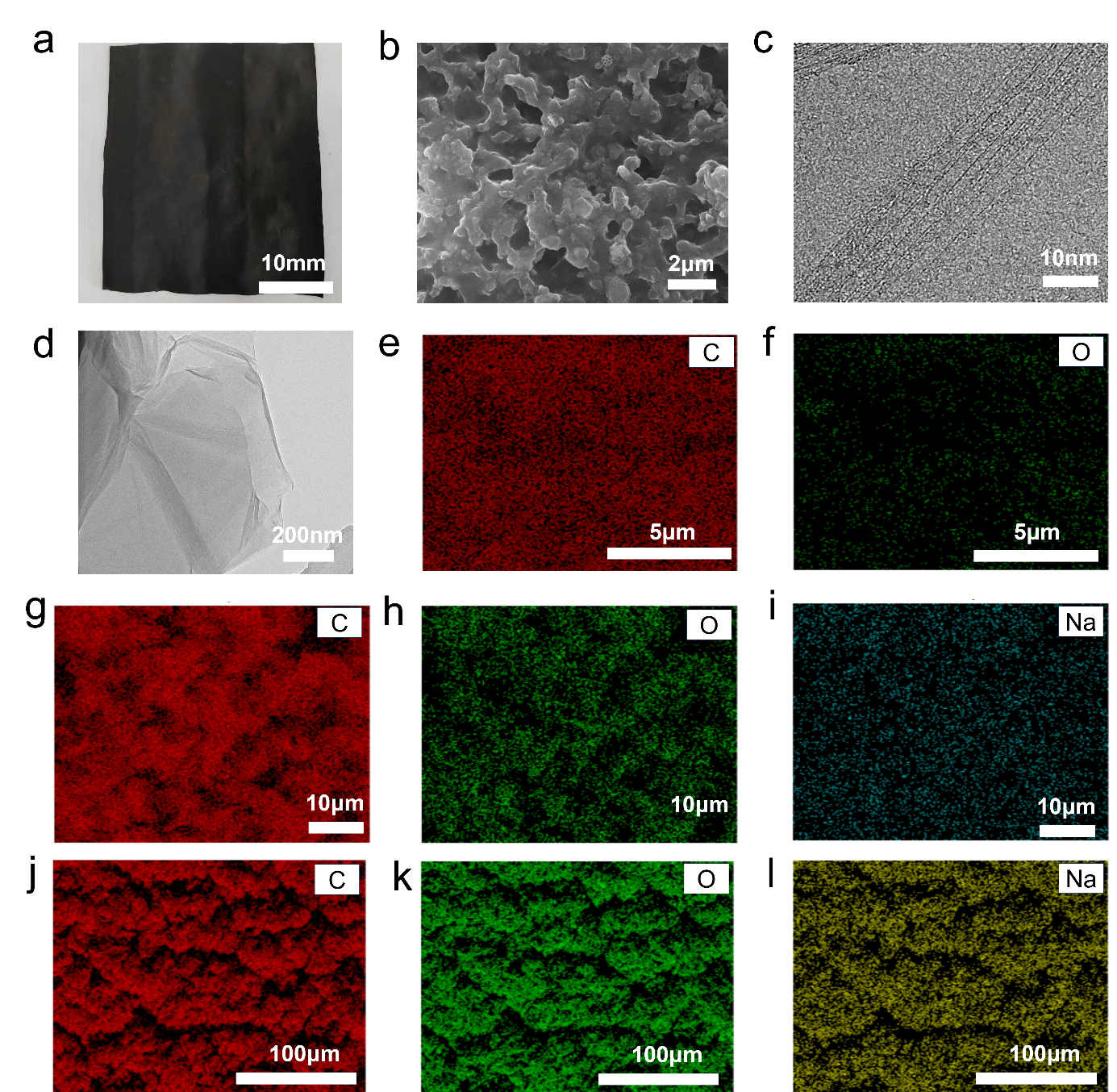


# Fig. S1 SEM and EDS images of the films. (a) Optical image of DSWCNT@latex, (b) SEM image of the latex substrate surface, (c) TEM image of SWCNT, (d) TEM image of GO, (e, f) EDS images of GO, (g-i) EDS images of SWCNT, and (j-l) EDS images of DSWCNT0.12


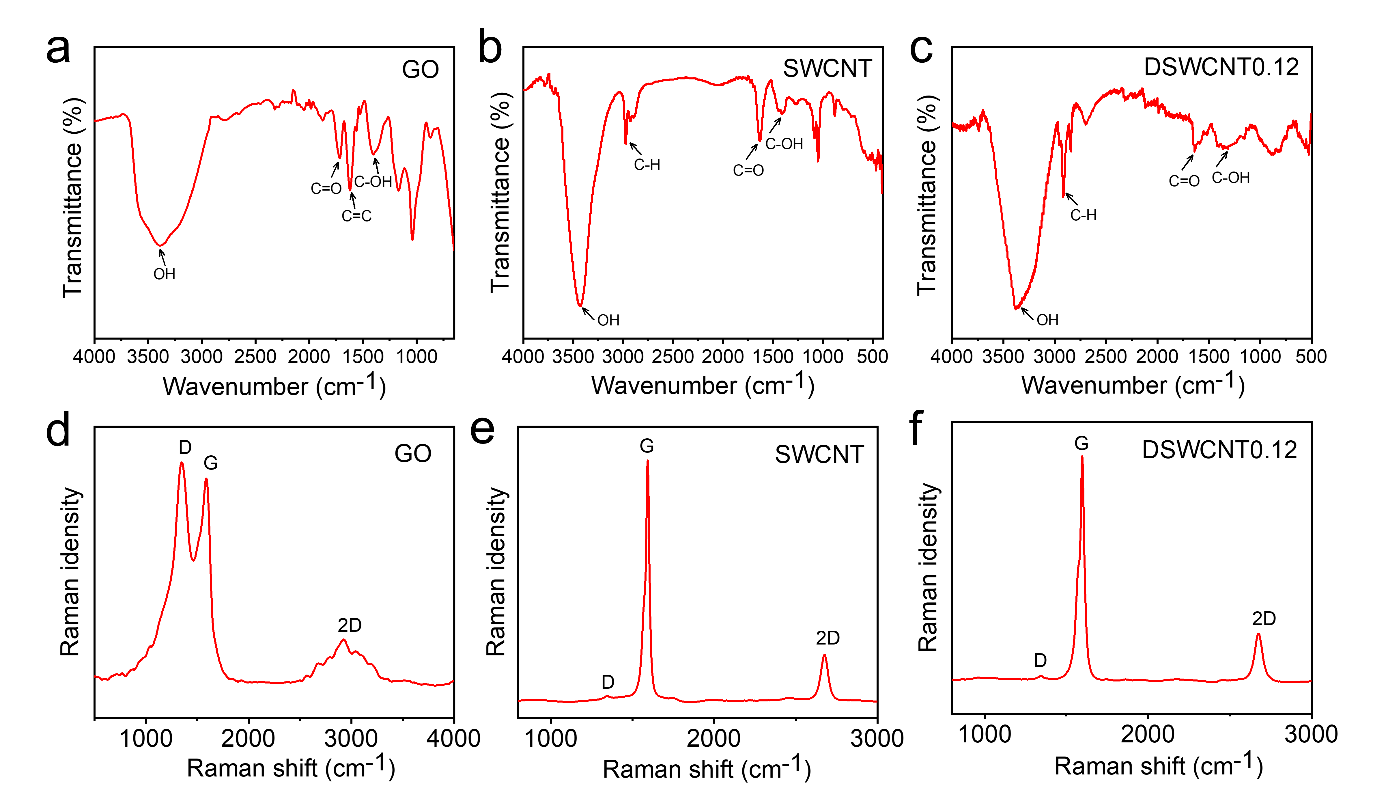


**Fig. S2** FTIR and Raman spectra of the films. (**a**) FTIR spectrum of GO film, (**b**) FTIR spectrum of SWCNT film, (**c**) FTIR spectrum of DSWCNT0.12 film, (**d**) Raman spectrum of GO film, (**e**) Raman spectrum of SWCNT film, and (**f**) Raman spectrum of DSWCNT0.12 film


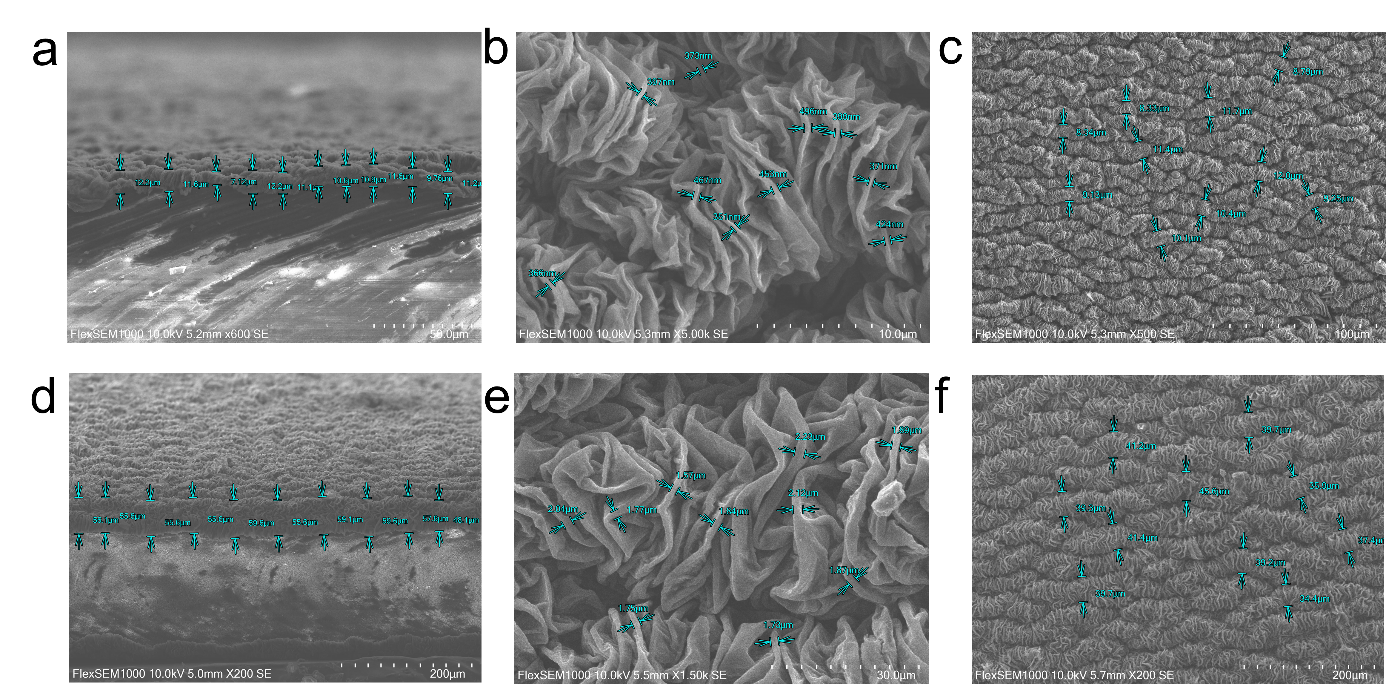


# Fig. S3 Wrinkling scales observed from SEM images. (a-c) SEM images of DSWCNT0.08 film, and (d-f) SEM images of DSWCNT0.12 film


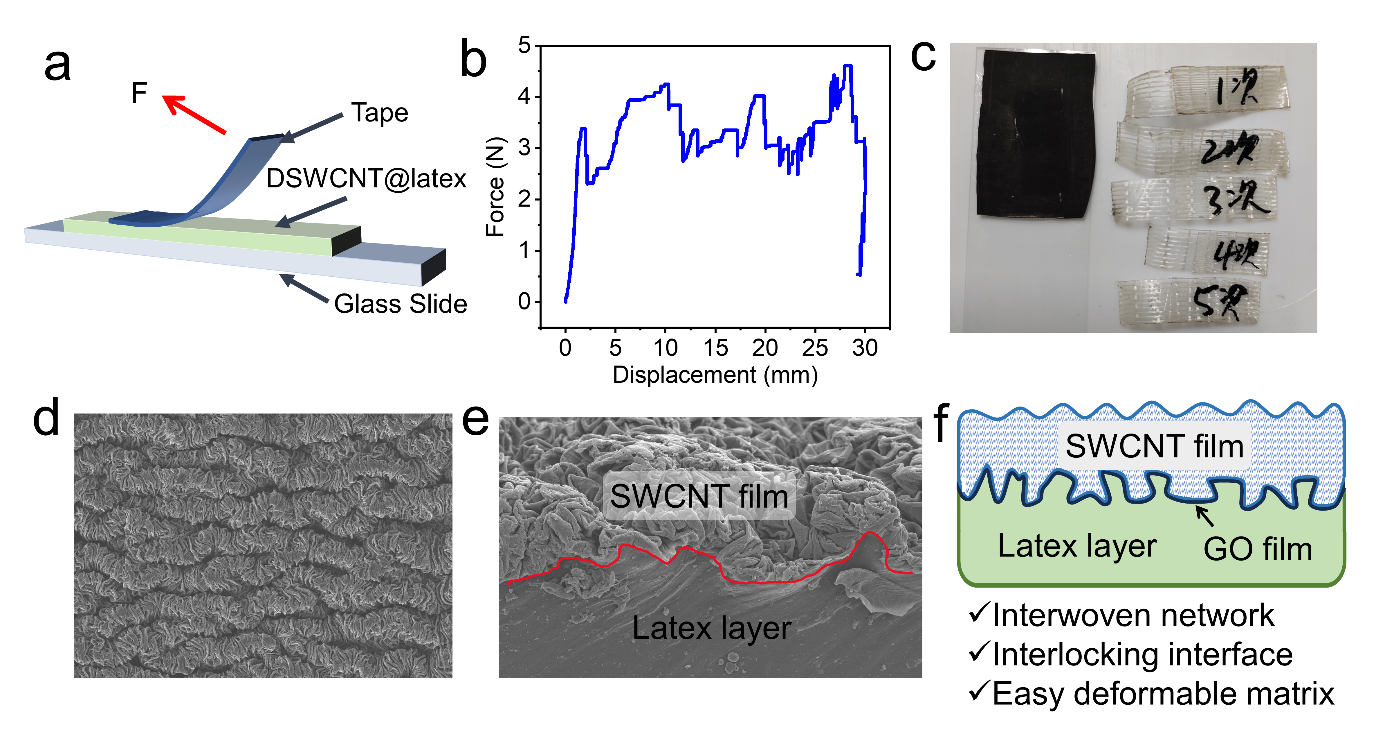


**Fig. S4** Mechancial performance of the tape stripping test. (**a**) Illustration of the tape stripping test, (**b**) tensile force of the tape stripping test, (**c**) optical image of the tape stripping test, (**d**) SEM image of DSWCNT0.12 film, (**e**) cross-sectional SEM image of DSWCNT0.12 film, and (**f**) cross-sectional illustration of the interlocking structure


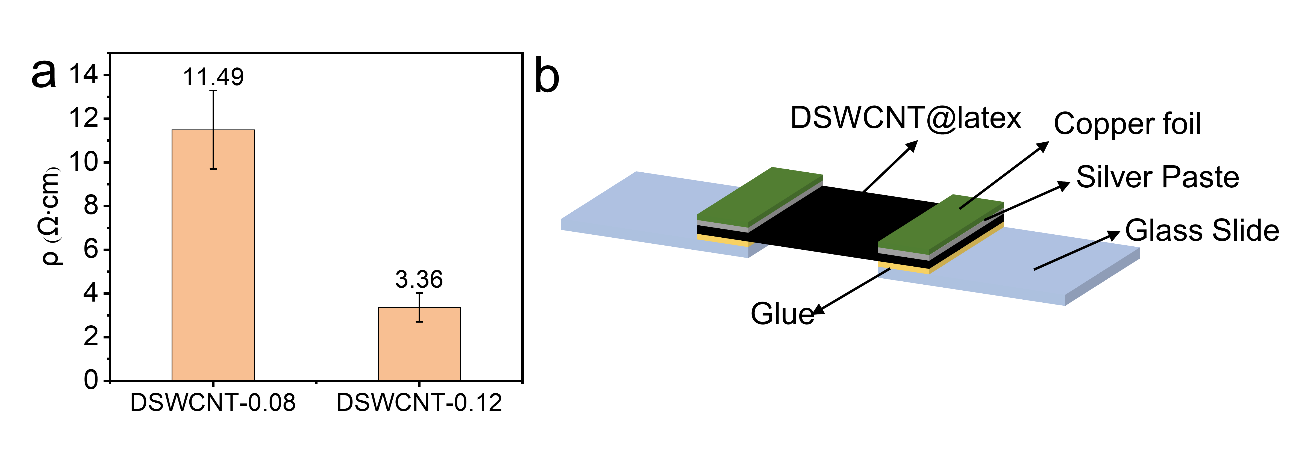


**Fig. S5** Resistivity of the films. (**a**) Average resistivity of DSWCNT0.08 and DSWCNT0.12 films, and (**b**) assembly diagram of strain sensor


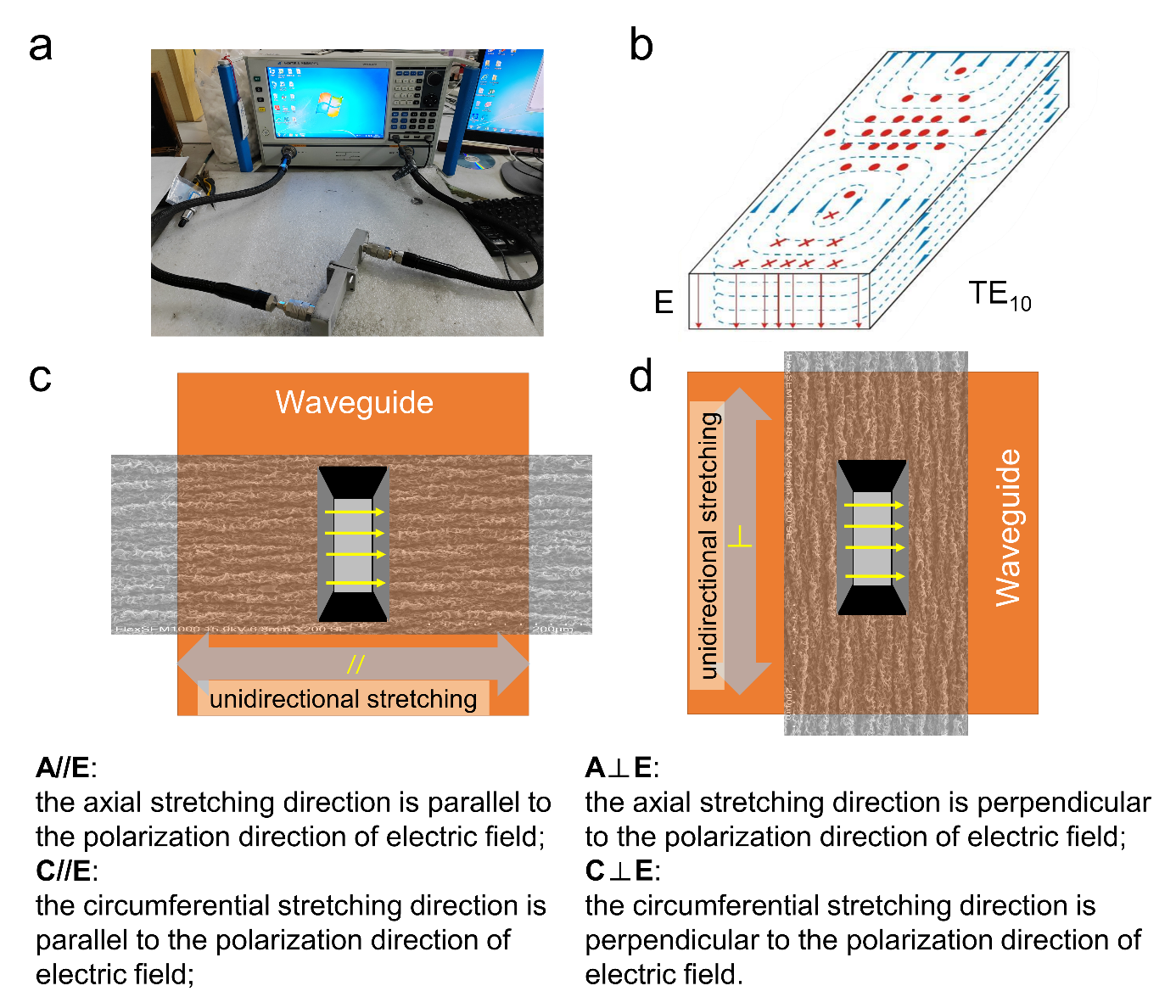


**Fig. S6** EMI shielding measurement illustration of the anisotropic films. (**a**) A vector network analyzer, (**b**) the mapping of TE_10_ transverse wave in the waveguide, (**c**) the stretching direction is parallel to the polarization direction of the electric field, including A//E and C//E, and (**d**) the stretching direction is perpendicular to the polarization direction of the electric field, including A⊥E and C⊥E


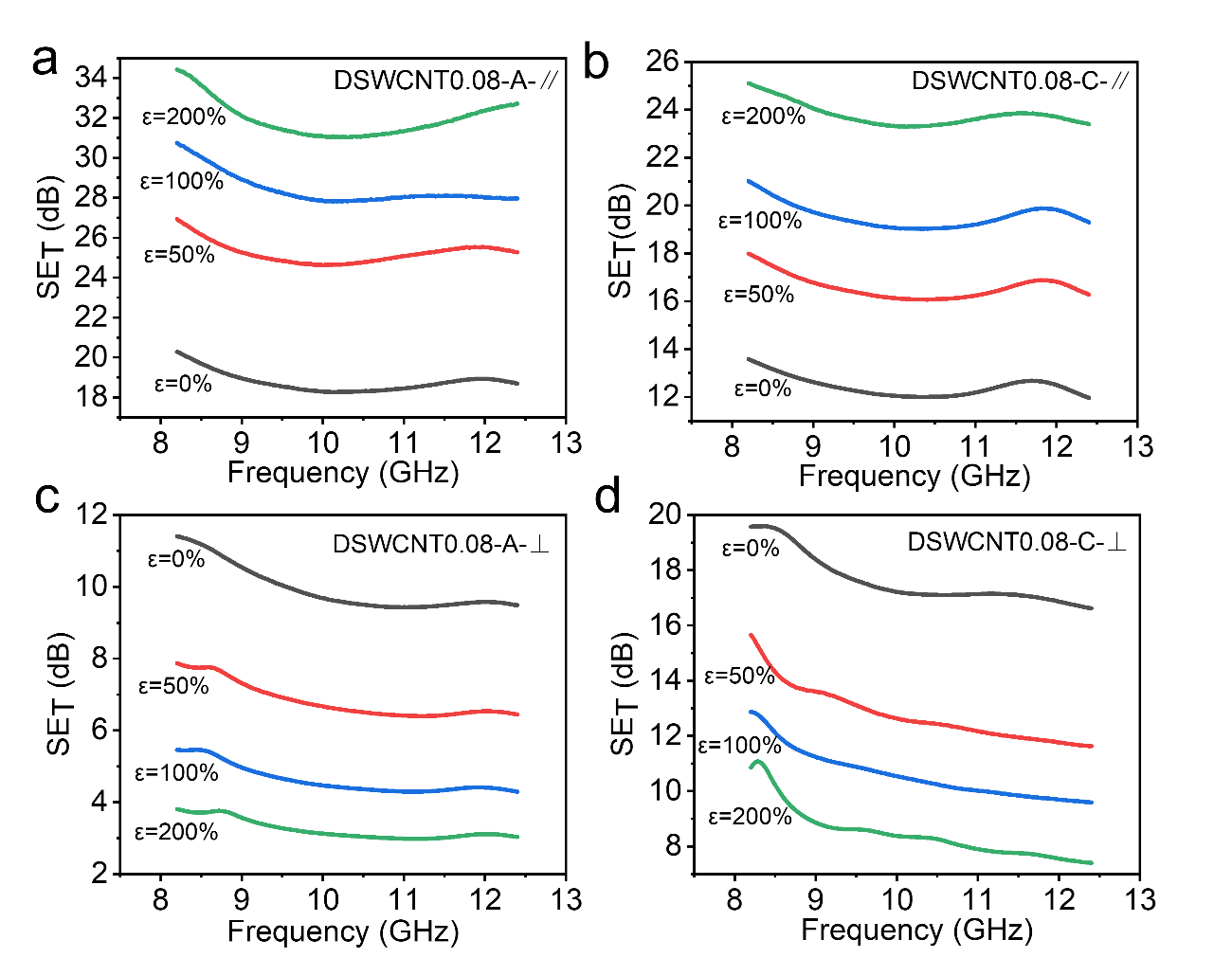


**Fig. S7** SE_T_ of the stretched DSWCNT0.08 film. (**a**) SE_T_ of the stretched DSWCNT0.08 film when A//E, (**b**) SE_T_ of the stretched DSWCNT0.08 film when C//E, (**c**) SE_T_ of the stretched DSWCNT0.08 film when A⊥E, and (**d**) SE_T_ of the stretched DSWCNT0.08 film when C⊥E


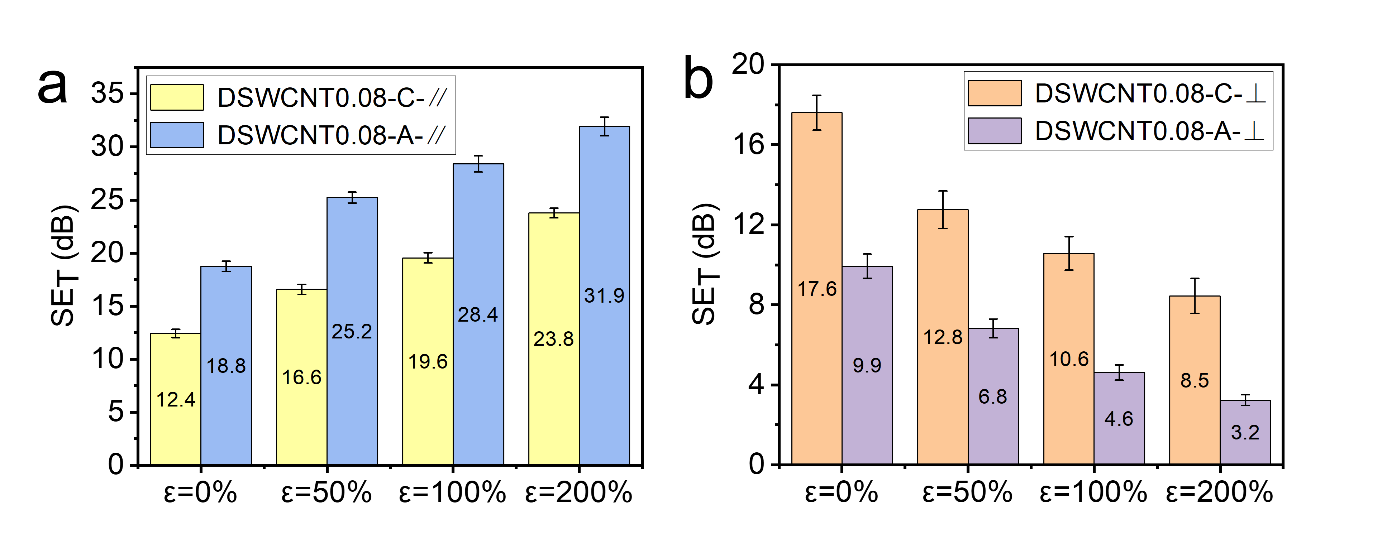


**Fig. S8** SE_T_ of DSWCNT0.08 film at different strains. (**a**) SE_T_ of DSWCNT0.08 film when the axial and circumferential drawing direction is parallel to the electric field direction, and (**b**) SE_T_ of DSWCNT0.08 film when the axial and circumferential drawing direction is perpendicular to the electric field direction


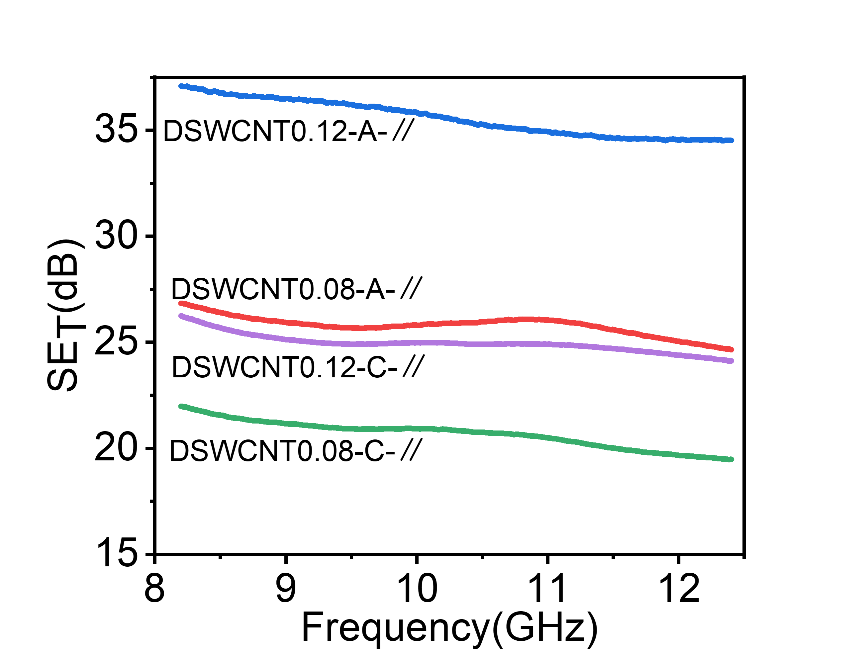


Fig. S9 Comparison of SE_T_ of the films without stretching (at 0% strain)

**Fig. S10** Cycling stability of DSWCNT0.12 film. (**a**) Comparison of SE_T_ of DSWCNT0.12 film before and after 10000 stretching cycles(A//E), (**b**) comparation of SE_T_ of DSWCNT0.12 film before and after punching(A//E), and (**c**) comparation of SE_T_ of DSWCNT0.12 film before and after vacuum impregnating with methyl silicone oil (A//E)


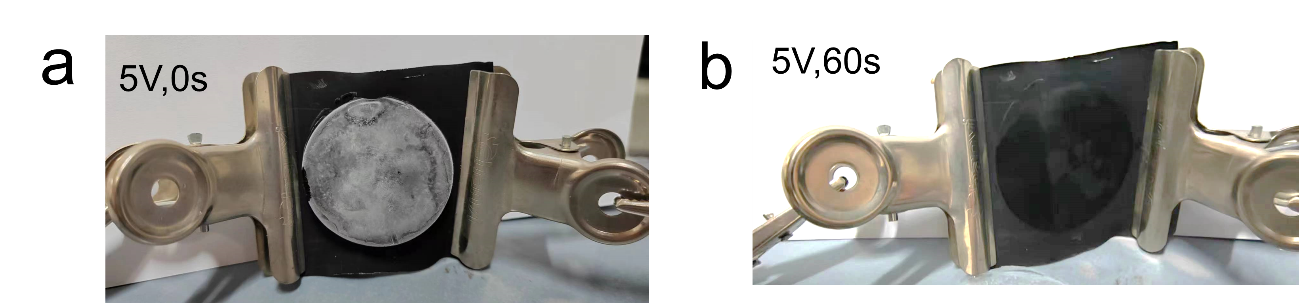


**Fig. S11** Deicing performance of DWSCNT0.12 film. Optical images of the deicing performance of DWSCNT0.12 film under 5V-heating at (**a**) 0 s and (**b**) 60 s


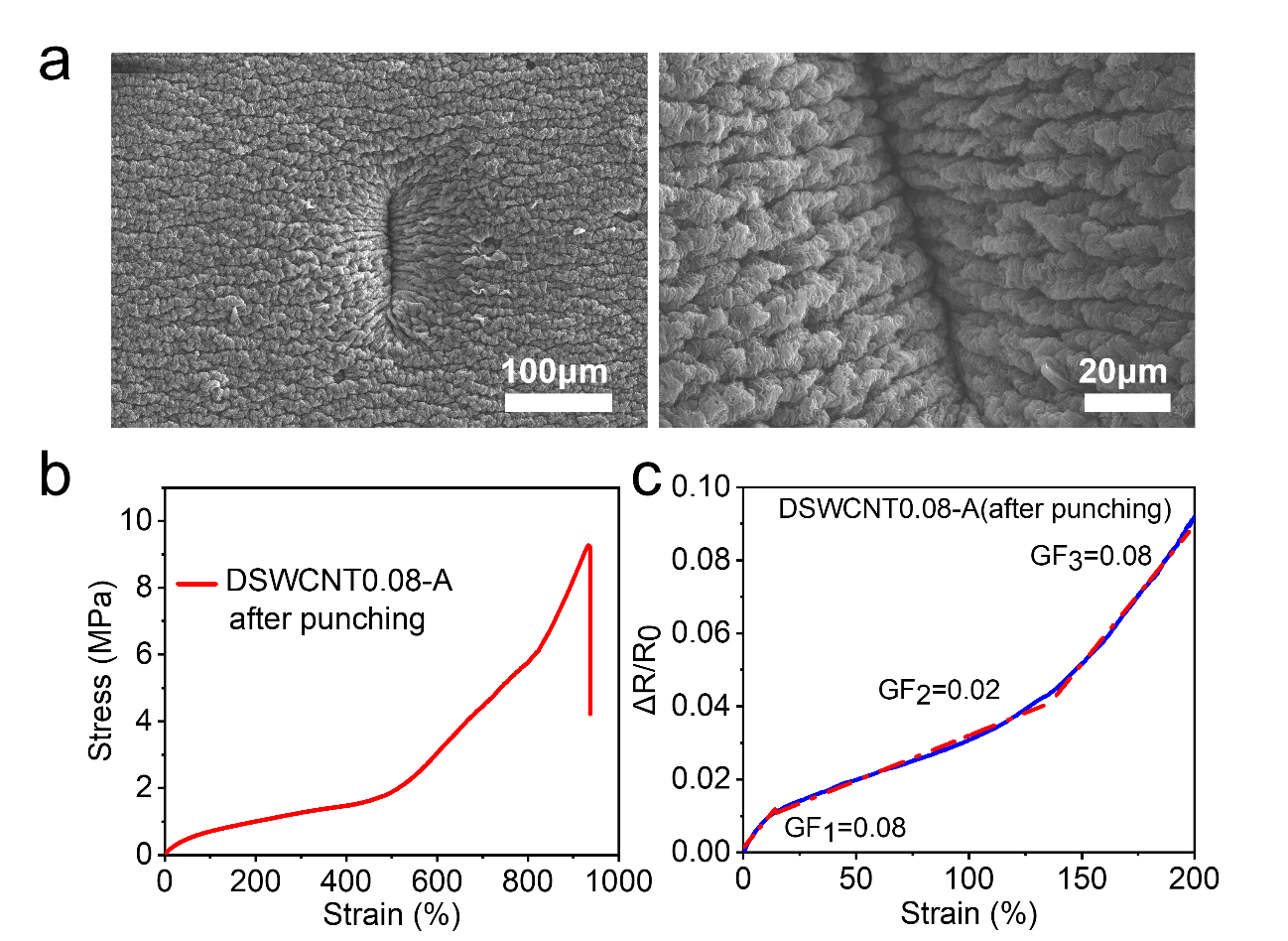


**Fig. S12** Mechanical property of the punched DSWCNT0.08 film. (**a**) SEM images of the punched DSWCNT0.08 film, on which a slit could be observed, (**b**) stress-strain curve of the punched DSWCNT0.08 film, (**c**) ΔR/R_0_-ε curve of the punched DWSCNT0.08 film during axial stretching


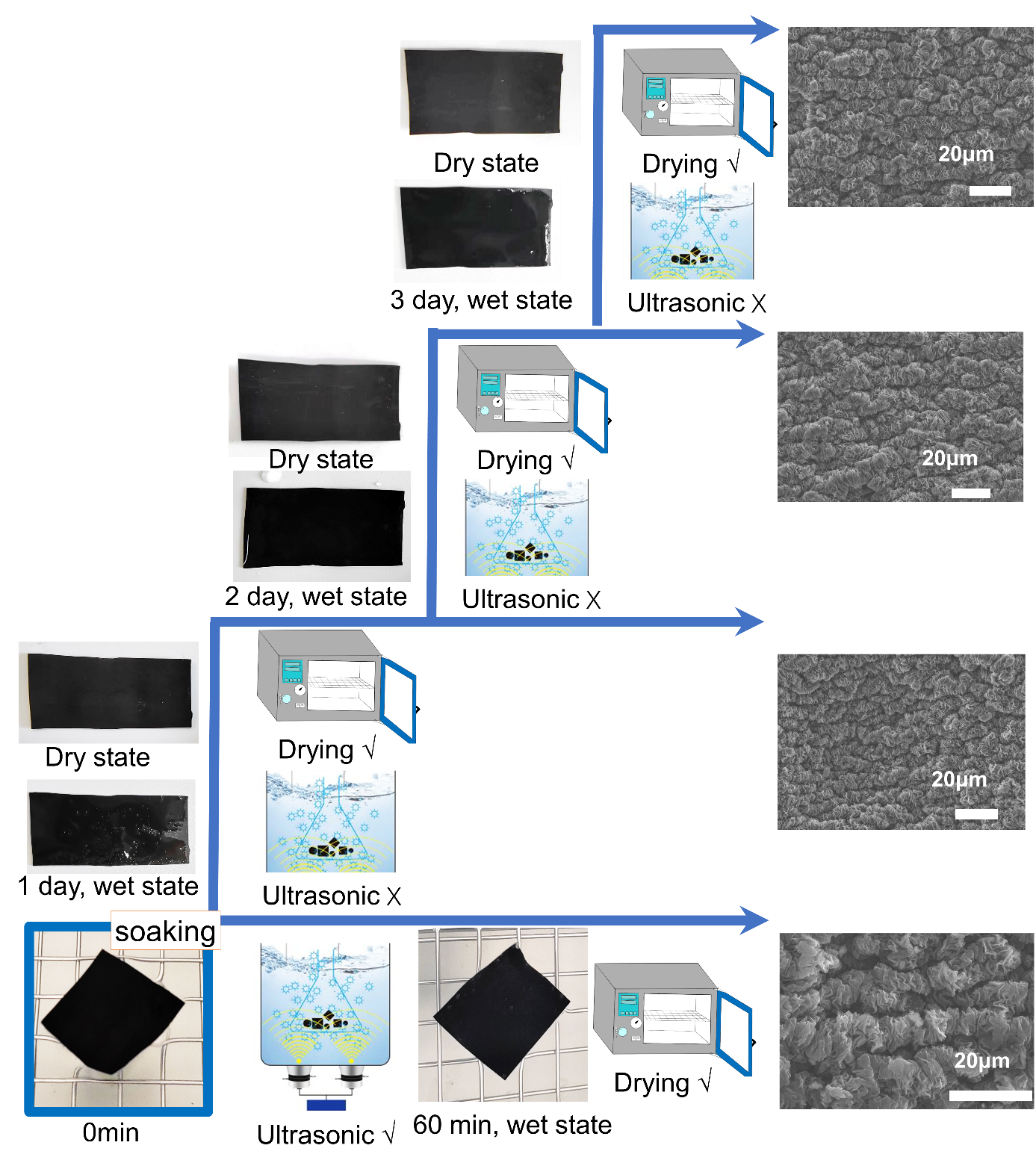


**Fig. S13** Water resistance performance of the films. The film could withstand 60min-soaking under ultrasonic treatment, and 3day-soaking without ultrasonic treatment


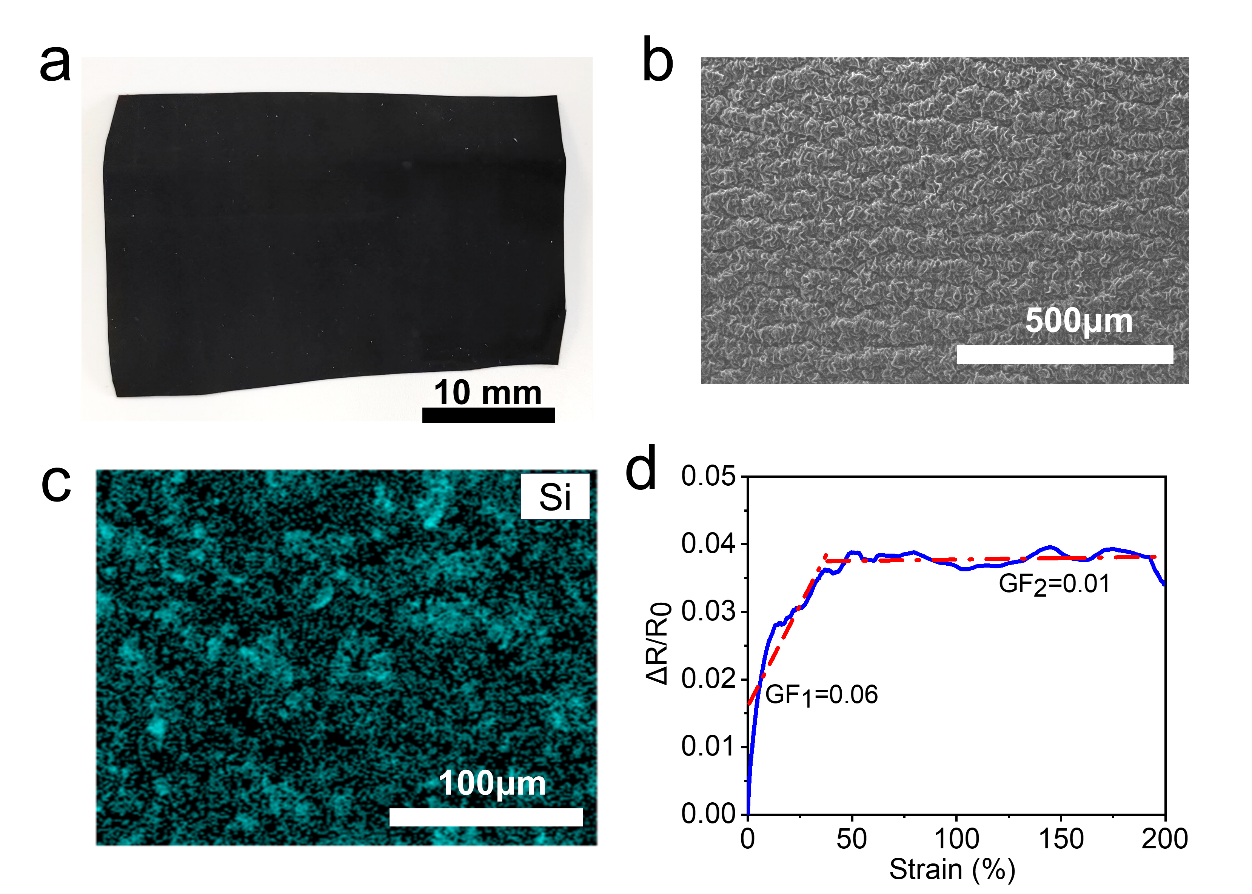


**Fig. S14** Microstructure of DSWCNT0.12 film impregnated with methyl silicone oil. (**a**) Optical image of DSWCNT0.12 film impregnated with methyl silicone oil, (**b**) SEM image of DSWCNT0.12 film impregnated with methyl silicone oil, (**c**) elemental distribution of Si on the surface of DSWCNT0.12 film impregnated with methyl silicone oil, (**d**) ΔR/R_0_-ε curve of DWSCNT0.12 film during axial stretching impregnated with methyl silicone oil

# Movie S1 Elephant trunk-inspired fabrication process

# Movie S2 Demonstration of EMI shielding effectiveness

# Movie S3 Recording Joule heating with an infrared camera

# Movie S4 Air bubbling using the punched film

# Movie S5 Rolling droplets on the film impregnated with methyl silicone oil

**Supplementary References**

1. L.-C. Jia, L. Xu, F. Ren, P.-G. Ren, D.-X. Yan et al., Stretchable and durable conductive fabric for ultrahigh performance electromagnetic interference shielding. Carbon **144**, 101–108 (2019). <https://doi.org/10.1016/j.carbon.2018.12.034>
2. F. Deng, J. Wei, Y. Xu, Z. Lin, X. Lu et al., Regulating the electrical and mechanical properties of tas_2_ films via van der waals and electrostatic interaction for high performance electromagnetic interference shielding. Nano-Micro Lett. **15**, (2023), <https://doi.org/10.1007/s40820-023-01061-1>
3. Y. Bai, B. Y. Zhang, G. Q. Fei, Z. L. Ma, Composite polymeric film for stretchable, self-healing, recyclable EMI shielding and Joule heating. Chem. Eng. J. **478,** 147382 (2023). <https://doi.org/10.1016/j.cej.2023.147382>
4. X. Jiang, J. W. Zhou, X. K. Zhong, Z. P. Hu, R. Hu et al., Stretchable PEDOT:PSS/Li-TFSI/XSB composite films for electromagnetic interference shielding. ACS Appl. Mater. Interfaces **15**, 8521-8529 (2023), <https://doi.org/10.1021/acsami.2c21604>
5. A. Katheria, P. Das, S. K. Ghosh, J. Nayak, K. Nath et al., Fabrication of 2D nanomaterial reinforced co-continuous binary blend composites for thermal management and EMI shielding applications. J. Polym. Res. **30**, (2023). <https://doi.org/10.1007/s10965-023-03843-y>
6. Z. F. Xu, J. L. Chen, G. Q. Wang, Y. Q. Zhao, B. Shen et al., Stretchable and translucent liquid-metal composite mesh for multifunctional electromagnetic shielding/sensing and Joule heating. Compos. Sci. Technol. **249**, 110512 (2024). <https://doi.org/10.1016/j.compscitech.2024.110512>
7. R. Zhu, Z. Li, G. Deng, Y. Yu, J. Shui et al., Anisotropic magnetic liquid metal film for wearable wireless electromagnetic sensing and smart electromagnetic interference shielding. Nano Energy **92**, 106700 (2022). <https://doi.org/10.1016/j.nanoen.2021.106700>
8. Z. Wang, X. Xia, M. Zhu, X. Zhang, R. Liu et al., Rational assembly of liquid metal/elastomer lattice conductors for high-performance and strain-invariant stretchable electronics. Adv. Funct. Mater. **32**, 2108336 (2022). <https://doi.org/10.1002/adfm.202108336>
9. J. Dong, S. Luo, S. Ning, G. Yang, D. Pan et al., MXene-coated wrinkled fabrics for stretchable and multifunctional electromagnetic interference shielding and electro/photo-thermal conversion applications. ACS Appl. Mater. Interfaces **13**, 60478–60488 (2021). <https://doi.org/10.1021/acsami.1c19890>
10. X. Hu, L. Mou, Z. Liu, Stretchable electromagnetic interference shielding and antenna for wireless strain sensing by anisotropic micron-steel-wire based conductive elastomers. Chin. Phys. B **30**, 018401 (2021). <https://doi.org/10.1088/1674-1056/abc2b3>
11. W. Chen, L.-X. Liu, H.-B. Zhang, Z.-Z. Yu Kirigami-inspired highly stretchable, conductive, and hierarchical Ti_3_C_2_T*_x_* MXene films for efficient electromagnetic interference shielding and pressure sensing. ACS Nano **15**, 7668–7681 (2021). <https://doi.org/10.1021/acsnano.1c01277>
12. Z. B. Chen, S. D. Yang, J. H. Huang, Y. F. Gu, W. B. Huang et al., Flexible, transparent and conductive metal mesh films with ultra-high FOM for stretchable heating and electromagnetic interference shielding. Nano-Micro Lett. **16**, (2024). <https://doi.org/10.1007/s40820-023-01295-z>
13. Y. Yu, P. Yi, W. Xu, X. Sun, G. Deng et al., Environmentally tough and stretchable MXene organohydrogel with exceptionally enhanced electromagnetic interference shielding performances. Nano-Micro Lett. **14**, 77 (2022). <https://doi.org/10.1007/s40820-022-00819-3>
14. Y. Feng, J. Song, G. Han, B. Zhou, C. Liu et al., Transparent and stretchable electromagnetic interference shielding film with fence-like aligned silver nanowire conductive network. Small Meth. **7**, 2201490 (2023). <https://doi.org/10.1002/smtd.202201490>
15. D. Feng, D. Xu, Q. Wang, P. Liu, Highly stretchable electromagnetic interference (EMI) shielding segregated polyurethane/carbon nanotube composites fabricated by microwave selective sintering. J. Mater. chem. C **7,** 7938-7946 (2019). <https://doi.org/10.1039/c9tc02311a>
